# Comparative genomics in “*Candidatus* Kuenenia stuttgartiensis” reveal high genomic plasticity in the overall genome structure, CRISPR loci and surface proteins

**DOI:** 10.1186/s12864-020-07242-1

**Published:** 2020-12-01

**Authors:** Chang Ding, Lorenz Adrian

**Affiliations:** 1 Helmholtz Centre for Environmental Research – UFZ, Environmental Biotechnology, Permoserstraße 15, 04318 Leipzig, Germany; 2 Chair of Geobiotechnology, Technische Universität Berlin, Ackerstraße 76, 13355 Berlin, Germany

**Keywords:** “*Ca.* Kuenenia stuttgartiensis”, Genome sequencing, Proteomics, CRISPR, Nitrite reductase, S-layer protein

## Abstract

**Background:**

Anaerobic ammonium oxidizing bacteria (anammox bacteria) are contributing significantly to the nitrogen cycle and are successfully used in wastewater treatment. Due to the lack of complete genomes in the databases, little is known about the stability and variability of their genomes and how the genomes evolve in response to changing environments.

**Results:**

Here we report the complete genome of the anammox bacterium “*Candidatus* Kuenenia stuttgartiensis” strain CSTR1 which was enriched planktonically in a semi-continuous stirred-tank reactor. A comparison of the genome of strain CSTR1 with the genome of “*Ca.* Kuenenia stuttgartiensis” MBR1 and the draft genome of KUST showed > 99% average nucleotide identity among all. Rearrangements of large genomic regions were observed, most of which were associated with transposase genes. Phylogenetic analysis suggests that strain MBR1 is more distantly related to the other two strains. Proteomic analysis of actively growing cells of strain CSTR1 (growth rate ~ 0.33 d^− 1^) failed to detect the annotated cytochrome *cd*_1_-type nitrite reductase (NirS) although in total 1189 proteins were found in the proteome. Yet, this NirS was expressed when strain CSTR1 was under stress or starvation (growth rate < 0.06 d^− 1^). We also observed large sequence shifts in the strongly expressed S-layer protein compared to other “*Ca.* Kuenenia” strains, indicating the formation of hybrids of genes encoding the surface proteins.

**Conclusions:**

“*Ca.* Kuenenia” strains appear to be relatively stable in their basic physiological traits, but show high variability in overall genome structure and surface proteins.

## Background

Anaerobic ammonium oxidation (anammox) is an important biological process that contributes significantly to the global nitrogen cycle [1], and is increasingly popular in state-of-the-art wastewater treatment due to its low energy consumption, low material cost, and small reactor footprint [2]. As described so far, the anammox process is catalyzed by anammox bacteria belonging to six different genera [3]. Yet, the dominant genera in waste water treatment plants are usually “*Candidatus* Kuenenia” or “*Ca.* Brocadia” [4–6].

Although “*Ca.* Brocadia anammoxidans” is the first reported anammox species [7], “*Ca.* Kuenenia stuttgartiensis” is by far the most extensively studied in terms of cell structure, physiology and biochemistry [8]. The first draft genome of an anammox bacterium was determined from a mixed community by metagenomics resulting in an almost complete (estimated 98%) genome of a “*Ca.* Kuenenia stuttgartiensis” strain (hereafter referred to as strain KUST) [9]. The KUST genome encoded a hypothesized anammox pathway and many other genes encoding versatile metabolic functions. Later, the roles of some key proteins encoded in the KUST genome were experimentally validated, e.g., hydrazine synthase (EC 1.7.2.7), hydrazine dehydrogenase (EC 1.7.2.8), hydroxylamine dehydrogenase (EC 1.7.2.6), and an S-layer protein [10–12]. Most notably, in 2011, it was confirmed by inhibitor/scavenger tests and fluorescence staining that in “Ca. Kuenenia” the key intermediate hydrazine was produced from nitric oxide instead of the previously speculated hydroxylamine [13]. In contrast, the functions of multiple large membrane-bound complexes are not yet functionally understood in detail [14]. For improved biochemical understanding of the anammox process, complete genome and protein expression data are crucial.

Currently, at least 37 anammox genome assemblies are reported (Table S1) with GC content values of 37 to 45% (median 41.1%), genome sizes of 2.3 to 5.2 Mbp (median 3.8 Mbp), and scaffold numbers of 1 to > 1000 (median 153). The “*Ca.* Kuenenia” genome KUST assembly was a high-quality draft containing five supercontigs which was sequenced in 2002 [9]. The reactor from which the KUST cells were obtained was maintained for years and an IonTorrent-based resequencing in 2012 confirmed the stability of the genome [12]. Interestingly, after only 2 years in 2014, another resequencing effort using the single-molecule real-time (SMRT) sequencing technique revealed the dominance of a new “*Ca.* Kuenenia stuttgartiensis” strain MBR1, while the original KUST strain was barely detectable. The genome of strain MBR1 was so far the only complete genome among all anammox bacteria [15]. Due to the availability of only one complete genome for anammox bacteria, it is currently not possible to make comparison on the genome structure or infer conserved genomic regions among anammox species.

In our previous work, we obtained granules from an anammox reactor treating landfill leachate at Beijing University of Technology. Initially, this reactor culture contained four anammox genera (“*Ca.* Kuenenia”, “*Ca.* Anammoxoglobus”, “*Ca.* Brocadia”, and “*Ca.* Jettenia”, all together representing 0.5% of the total population). After three transfers in serum bottles the culture contained only a single anammox genus (“*Ca.* Kuenenia”) at 17% of the total population and became granule-free [16]. This planktonic culture was then transferred to a semi-continuous stirred-tank reactor (CSTR), and exhibited stable growth rate of up to 0.33 d^− 1^ and high anammox activity [17]. Illumina-based amplicon sequencing of 16S rRNA genes and fluorescence in-situ hybridization revealed the dominance of “*Ca.* Kuenenia” (87% of the total population) [17].

In our present study, we determined the complete genome sequence of the dominant “*Ca.* Kuenenia” species from our CSTR reactor and refer to it as strain CSTR1. The genome of strain CSTR1 is the second complete anammox genome to date, and unveils how variable the genomes are among the closely related “Ca. Kuenenia” strains. CRISPR analysis also hints at the strain differentiation history among these strains. Finally, information was obtained on possible functions of key genes in the anammox genome via expression analyses using shotgun proteomics.

## Results

### Complete genome of “*Ca.* Kuenenia stuttgartiensis” strain CSTR1

The longest contig assembled from SMRT sequencing of the CSTR reactor effluent had a coverage of ~ 93 and a length of 4.3 Mbp (Table S2). All other contigs had a coverage of < 35 and a size of < 55 kbp, and were mostly affiliated with *Methyloversatilis*, consistent with amplicon sequencing results of the same reactor (5 months before the sampling for genome sequencing) showing *Methyloversatilis* as the second most abundant population in the community [17].

The longest contig of 4.3 Mbp was circularized and was found to contain a single 16S rRNA gene sequence which shared 100% identity with those of “*Ca.* Kuenenia stuttgartiensis” strains MBR1 and KUST over 1577 bp. Therefore, the circularized contig was confirmed to be the genome of strain CSTR1. The genome had a size of 4,334,932 bp, with a GC content of 41.03% (Table 1). Three copies of *dnaA* genes were found in the genome, and one of the three *dnaA* genes (locus tag KsCSTR_00010) was chosen as the origin of replication (ori) on the genome based on the cumulative GC skew graph (Fig. S1C). This *dnaA* is the only *dnaA* gene that has *dnaN* (KsCSTR_00020) and *gyrB* (KsCSTR_00040) genes at its immediate proximity. The genome sequence was deposited in the National Center for Biotechnology Information (NCBI) database (BioProject: PRJNA603163; BioSample: SAMN13921532; reads archive number: SRR11213620; genome accession number CP049055).

**Table 1:** Comparison of the genomes of “*Ca.* Kuenenia stuttgartiensis” strain KUST, strain MBR1, and strain CSTR1

Strain	KUST [9]	MBR1 [15]	CSTR1 (this study)
Status	High quality draft (5 supercontigs)	Complete genome	Complete genome
Size	4,218,325	4,406,132	4,334,932
Sequencing technology	Sanger sequencing	SMRT sequencing	SMRT sequencing
GC %	41.0	41.1	41.0
Prediction of coding sequence	dps/orpheus, AMIGene, glimmer/rbsfinder and genemarks/genemark. hmm	Prodigal [18] in Prokka pipeline [19]	AMIGene in MaGe pipeline [20]
# Coding sequences (CDSs)	4664	4043	4965
Coding density %	85.9	81.2	85.7
Average gene length	776	902	751
# rRNA operons (5S, 16S, 23S)	1	1	1
# tRNAs	44	45	45

MBR1 has much fewer predicted CDSs than the other two genomes and therefore a lower coding density and a higher average gene length due to usage of a different coding sequence prediction tool (Prodigal)

### Structural variations among “*Ca.* Kuenenia” strains as revealed by genome comparison

We compared the overall genome structures of strain CSTR1 and strain MBR1 as these are the only two available complete anammox genomes to date. The cumulative GC skew graph of the MBR1 genome (Fig. S1A) suggested that KSMBR1_2151 is the *ori*-defining *dnaA* gene, which is in contrast to its original description. The use of KSMBR1_2151 as the *ori*-defining *dnaA* gene was further supported by the high sequence similarity of KSMBR1_2151 with KsCSTR_00010 defining the *ori* in strain CSTR1 and the vicinity of *dnaN* and *gyrB*. To facilitate the comparison between strain MBR1 and strain CSTR1, we generated a reverse complement of the genome sequence MBR1, chose the first base pair of KSMBR1_2151 as position 1, and called the converted genome MBR1b (GC skew as shown in Fig. S1B).

**Fig. 1:**
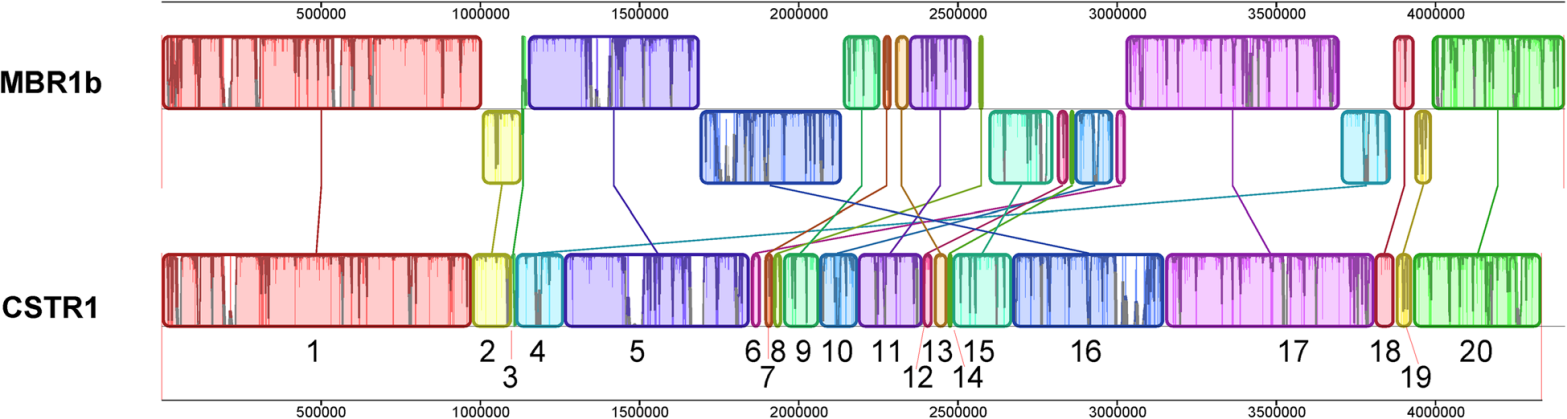
Visualized genome alignment of the genome of “*Ca.* Kuenenia stuttgartiensis” strain CSTR1 and MBR1b. In total, 20 locally collinear blocks (LCBs) were identified by Mauve and labelled in the figure. Histograms within the LCBs indicate sequence similarity within the regions

Mauve alignment of the two circularized genomes MBR1b and CSTR1 showed strong synteny between the two genomes, dividing the genome sequences into 20 conserved regions, referred to as ‘locally collinear blocks’ (LCB) (Fig. 1). LCB 1 and 20 (combined) are syntenic and located around the ori (~ 1.4 Mbp in length). Also LCB 5 (~ 0.6 Mbp in length, containing the rRNA gene operon) and LCB 17 (~ 0.7 Mbp in length) are large LCBs conserved in orientation and genome position. Considerable rearrangements were found between many other LCBs of genome MBR1b and strain CSTR1 (Fig. 1). A total of 11 reversal steps were necessary in order to convert between the genome of strain CSTR1 and the genome MBR1b as calculated using GRIMM (Table S3). A close examination of the flanks of the rearranged genomic blocks almost always identified transposase genes corroborating genomic motility (Table S4).

### Phylogenetic analyses of the three “*Ca.* Kuenenia stuttgartiensis” strains showing strain MBR1 is phylogenetically more distant

The three genomes KUST, MBR1 and CSTR1 are highly similar, as indicated by high average nucleotide identity (ANI) values and tetranucleotide signature correlations (Table 2). ANI values showed that strain KUST and strain CSTR1 are slightly closer with each other than with strain MBR1. In terms of rRNA gene sequences, strain KUST and strain CSTR1 are 100% identical in 5S–16S-23S rRNA concatenated sequences, while MBR1 has one mismatch at 23S rRNA. We also employed concatenated conserved protein analysis [9] to obtain a higher resolution in phylogenetic relationship among the strains. In the alignment with a length of 31,842 nt we counted the number of nucleotide positions where one of the strains had a different nucleotide while the other two had the same nucleotide, suggesting a mutation in the strain with the nucleotide divergence. This analysis gave 2 nucleotide changes in KUST, 31 in MBR1, and 4 in CSTR1. When doing the same analysis with amino acid sequences of the concatenated protein sequences (10,564 aa length) we found 8 changes in MBR1, and none in the other two strains. Therefore, based on the analyses of ANI, rRNA sequences, and concatenated conserved proteins, strain MBR1 is phylogenetically more distant from strain KUST and strain CSTR1.

**Table 2:** Genome sequence similarity among the genomes of “*Ca.* Kuenenia stuttgartiensis” KUST, strain MBR1, and strain CSTR1

	KUST	MBR1	CSTR1
**KUST**		Tetra 0.9981	Tetra 0.9985
**MBR1**	ANI 0.9940		Tetra 0.9994
**CSTR1**	ANI 0.9960	ANI 0.9922	

ANI - average nucleotide identity between genomes as calculated using the algorithm OrthoANIu [21, 22]

Tetra - correlation of tetranucleotide signatures between genomes as calculated using the program Tetra v1.02 [23]

The “*Ca.* Kuenenia” strain from which the KUST genome was obtained in 2002 was re-sequenced in 2012 [12] (hereafter KUST2012). We assembled the IonTorrent reads of KUST2012 (Additional file 1). There was a total of six mutations in concatenated conserved gene sequence in KUST2012 compared to KUST, but all were either insertion or deletion that caused frame shifts and therefore were probably sequencing or assembly errors. Therefore, there were probably no changes in gene sequences of the analyzed conserved proteins after ten years cultivation of KUST.

### Analysis of a shared CRISPR locus suggesting strain KUST might have differentiated earlier among the three strains

**Fig. 2:**
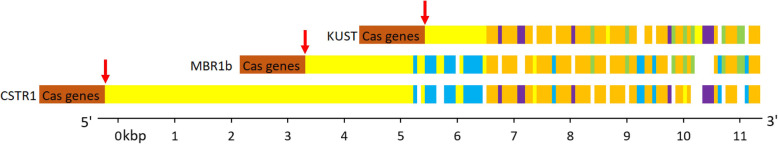
Visualized alignment of the largest CRISPR locus in “*Ca.* Kuenenia stuttgartiensis” genomes KUST, MBR1b, and CSTR1. The CRISPR locus in KUST2012 is almost identical to that in the KUST, except that the 31^st^ and 32^nd^ spacers were not present in KUST2012 (counting from the leader sequence end) and is therefore not shown. Orange: identical spacers among the three genomes; Blue/Green/Purple: identical spacers between two of the three genomes; Yellow: unique spacers; White: gap in the alignment; Brown: type I-B Cas gene clusters. These Cas gene clusters span a length of ~ 6 kbp and are not drawn to scale. Arrows indicate the location of a 138-bp leader sequence

All three “*Ca.* Kuenenia” genomes contain several CRISPR elements and CRISPR associated genes (Cas) (Table S5). Among these CRISPR elements one large CRISPR locus with more than 5 kbp and 80 or more spacers near a type I-B Cas gene cluster are shared by all three genomes (Table S6, Fig. 2). This type I-B CRISPR locus (coordinate: 1.18 Mbp in CSTR1, 3.78Mbp in MBR1b) located within LCB 4 which was flipped and relocated when comparing the genomes of CSTR1 and MBR1b (Fig. 1). The 30 bp repeat of the CRISPR locus is conserved among all three genomes but has no perfect match in the NCBI nt database to other genomes. Matches to the repeat sequence with an e-value of 0.12 were present to CRISPR loci in many other genomes, indicating only distant relationship. None of the spacer sequences (210 spacers in total) had a 100% match in the NCBI nt database other than from the three genomes themselves. 45 spacers are shared by all three “*Ca.* Kuenenia” genomes and all are located at the 3′ end of the CRISPR locus (Fig. 2). In total, 19, 35, and 85 spacers are unique to the genomes of strain KUST, strain MBR1, and strain CSTR1, respectively, and most of these unique spacers are located between the leader sequence (arrow in Fig. 2) and the common spacers. It is known that newly acquired spacers are inserted at the leader end of the CRISPR locus [24, 25]. Spacers seem to pop out at random locations (gaps in Fig. 2), the cause of which is unknown. Apart from the 45 common spacers (orange colored in Fig. 2), strain MBR1 and strain CSTR1 also shared 12 more spacers (blue colored in Fig. 2) that are located next to the 45 common spacers. This suggested that strain KUST might have differentiated first from the common ancestor of the three strains, and the common ancestor of strain MBR1 and strain CSTR1 continued to acquire the blue-colored spacers before they eventually diversified into two strains. We also examined changes in the CRISPR locus in KUST2012 compared to the original KUST genome A nearly identical locus was found in Node 4 of the KUST2012 assembly (Additional file 1) with 78 spacers. Compared to the CRISPR locus in the original KUST genome, two spacers (the 31^st^ and 32^nd^ counting from the leader end) were absent while no new spacers were found.

### Overview of the annotated genome of strain CSTR1

The annotated genome of strain CSTR1 contains 4965 protein-coding genes, one ribosomal RNA operon (5S, 16S, and 23S), one transfer-messenger RNA gene, and 45 tRNA genes (Table 1). The genome of strain CSTR1 contains genes encoding all described ribosomal proteins [26] except the two non-essential proteins L30 and L34. A total of 2849 genes encoding proteins of unknown functions were found, making up 57% of the total genes. Of the 4965 protein-coding genes in strain CSTR1, 4557 genes (92%) have homologs in strain KUST, 4215 genes (85%) have homologs in strain MBR1, and 4076 genes (82%) are found in both strain KUST and strain MBR1, based on a sequence identity threshold of 40% (homologs include matches with six-frame translations of genome sequences of strains KUST and MBR1) (Table S7). Pangenome analysis based on re-annotated genomes of strain KUST, MBR1, and CSTR1 showed that 2966 genes were shared by all three genomes, and numbers of specific genes are 122, 281, and 186, respectively (Fig. S2).

The genome of strain CSTR1 contains all reported essential genes related to anammox metabolism, including two identical gene sets for the heterotrimeric hydrazine synthase (KsCSTR_28210 / KsCSTR_28190 / KsCSTR_28200 and KsCSTR_12690 / KsCSTR_12670 / KsCSTR_12680), hydrazine dehydrogenase genes KsCSTR_46980 / KsCSTR_11820, hydroxylamine dehydrogenase gene KsCSTR_43280, and the alpha, beta and gamma subunits of nitrite:nitrate oxidoreductase (KsCSTR_08000 / KsCSTR_07970 / KsCSTR_07960).

Just as strain KUST and strain MBR1, strain CSTR1 also contains four different types of ATPase genes, three complex III genes, a complete set of genes encoding the reductive acetyl-CoA pathway for CO_2_ fixation, and two sets of complex I genes (both containing 14 subunits: A to N including the peripheral NADH input module *nuoEFG*) (KsCSTR_25990-KsCSTR_26120 and KsCSTR_45490-KsCSTR_45710). The complex I gene cluster KsCSTR_45490-KsCSTR_45710 was speculated to couple NADH oxidation to CO_2_ and menaquinone reduction [27]. The type 3b (sulf) hydrogenase operon in strain MBR1 (KSMBR1_3671–3674), which is absent in the genome KUST, is present in strain CSTR (KsCSTR_28360–28,390). This operon was speculated to be involved in hydrogen metabolism [15].

The abundance of transposase genes in the genome of strain CSTR1 as well as in strain KUST and strain MBR1 (> 5% of all genes) is significantly higher than average abundance (1.1%) in prokaryotic genomes [28], with > 200 full length or remnant transposase genes identified (Table S8, Table S9). Although there are as many as 38 groups of transposase genes in the three anammox genomes, the top 10 groups account for > 60% of all transposase genes. Interestingly, the group 13 transposase, which is a IS1634 family transposase, exists in 20 full copies in strain MBR1, but is absent in both strain KUST and strain CSTR1.

### Overall expression analysis of genes in strain CSTR1 by shotgun proteomics

The expressed proteome of strain CSTR1 was evaluated using triplicate samples from a semi-CSTR reactor running at 3 days hydraulic retention time (HRT). In total, 20,800 PSMs were found, leading to 5429 identified peptides and 1189 proteins, of which 1168 were quantified. The most abundant proteins in the proteomes were similar to those found in previous studies (Table S7) [14, 29], including most of the key enzymes involved in the anammox pathway such as hydrazine synthase, hydrazine dehydrogenase, nitrite:nitrate oxidoreductase, and hydroxylamine oxidase (Table S7). The genome of strain CSTR1 encodes two highly similar hydrazine dehydrogenase, KsCSTR_46980 (kustc0694, KSMBR1_2369) (gene tags in brackets are homologs in strains KUST and MBR1, same below) and KsCSTR_11820 (kustd1340, KSMBR1_1220). Abundances of unique peptides belonging to KsCSTR_46980 indicated that KsCSTR_46980 was highly expressed (Table S10), agreeing with the previous finding [11]. A nitrite transporter KsCSTR_14610 (kuste3055, KSMBR1_1070) (0.019%, 441) (abundance, abundance rank; same for further data given below) and an ammonium transporter KsCSTR_43840 (kustc1009, KSMBR1_2627) (0.0039%, 790) were expressed, while the previously characterized ammonium sensor KsCSTR_21800 (kuste3690, KSMBR1_3866) [30] was not found in the proteome.

Expression of putative respiratory complexes was similar to what was reported before, including the expression of complex I genes, ATPase genes, and Rieske/cytb complex genes [14]. Although the reactor was running at an HRT of 3 days and therefore cells were actively dividing, the previously reported division ring protein KsCSTR_10780 (kustd1438, KSMBR1_1135) [31] was not found in the proteome. One of the CRISPR-associated genes (KsCSTR_13270) near the large CRISPR locus described above was expressed at a level of 0.10% of the whole proteome (rank 134). The group 15 transposase KsCSTR_47830 (rank 1021) is the only transposase that is detected in the proteome.

One of the top 50 abundant proteins KsCSTR_28510 (KSMBR1_3708) (0.54% of total proteins, rank 27), annotated as a hypothetical exported protein, was not found in the genome of KUST including its six-frame translation, while all the other top 50 abundant proteins had homologs in the genome of strain MBR1 and KUST.

### Expression of putative nitrite reductase genes

The identity of the nitrite reductase gene in “*Ca.* Kuenenia” genomes is unclear. It was initially hypothesized that the annotated cytochrome *cd*_1_-type nitrite reductase (NirS) kuste4136 (KSMBR_0452 / KsCSTR_33370) converts nitrite to nitric oxide – a key step in the anammox metabolism [13]. However, a further study indicated that this NirS was barely detectable in the proteome of “*Ca.* Kuenenia” [14], while others detected NirS with as much as 28% sequence coverage [29]. This led to the question if the product of kuste4136 is the actual nitrite reductase, or if there might be another enzyme with nitrite reductase activity. Candidates for such an enzyme include two putative hydroxylamine oxidoreductase genes (KsCSTR_49490 / KSMBR1_2163 / kustc0458 and KsCSTR_29630 / KSMBR1_3792 / kuste4574) [27].

In our proteome datasets that came from samples taken from the actively running reactor at 3 d HRT, the three subunits of hydrazine synthase and hydrazine dehydrogenase represented the four most abundantly expressed proteins. Also the three subunits of nitrite:nitrate oxidoreductase and the two putative hydroxylamine oxidoreductases KsCSTR_49490 (1.19%, rank 14) and KsCSTR_29630 (0.35%, rank 46) were among the most abundant proteins in the proteome. In contrast, no NirS (KsCSTR_33370) was detected. Only when we examined multiple sets of whole proteome data over a period of almost 3 years (all were planktonic cells), we found that NirS appeared in some datasets (Table S11), all at relatively long HRTs (≥15d). The inconsistent detection of NirS suggested that it was at least dispensable for growth and energy conservation in “*Ca.* Kuenenia” strains. The other two nitrite reductase candidates KsCSTR_49490 and KsCSTR_29630 were consistently expressed in all examined datasets (Table S11).

### Sequence evolution of the gene encoding the S-layer protein

The gene kustd1514 in the genome KUST was characterized as a heavily glycosylated protein forming the S-layer of the bacterium [12, 32]. The homologs of this gene in strain MBR1 and strain CSTR1 are KSMBR1_1301 and KsCSTR_09970, respectively, both are located within LCB 1 (coordinate: 0.91 Mbp in CSTR1 and 0.94 Mbp in MBR1b). Just as kustd1514, KsCSTR_09970 was also highly expressed in the genome of strain CSTR1 (Table S7, rank 8). Interestingly, while KSMBR1_1301 shares 99% amino acid identity with kustd1514, KsCSTR_09970 shares only 53% identity with kustd1514. In fact, in the top 50 abundant proteins in strain CSTR1, all the other proteins exhibited > 95% amino acid sequence identity with their homologs in the genome of strain MBR1 and KUST (Table S7).

**Fig. 3:**
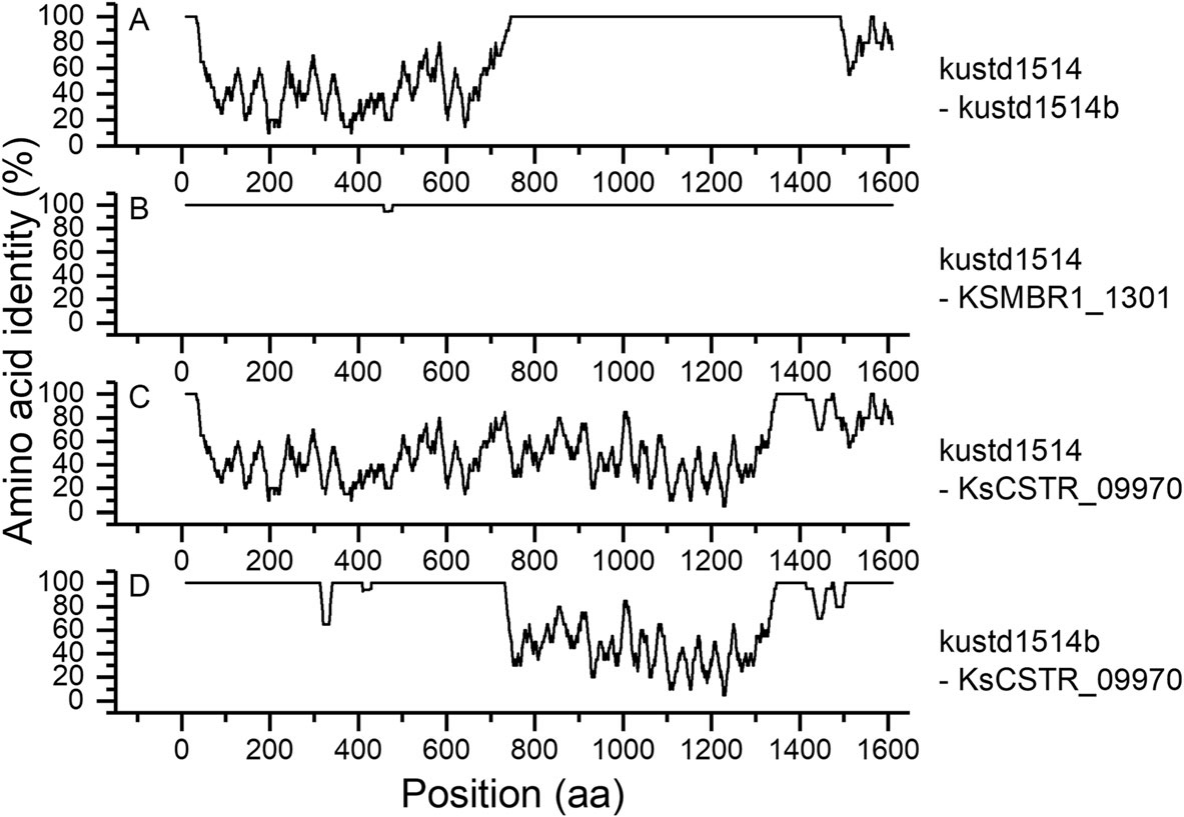
Amino acid sequence identity comparison among the four S-layer protein homologs in KUST (kustd1514), KUST2012 (kustd1514b), strain MBR1 (KSMBR1_1301), and strain CSTR1 (KsCSTR_09970). Shown are always comparisons of the two sequences indicated on the right side. Positions (aa) refer to the amino acid (aa) positions in the alignment of the four proteins. Window size: 20 amino acids. Step: 1 amino acid

The re-sequenced KUST genome in 2012 [12] revealed a kustd1514 with significantly changed amino acid sequence (hereafter referred to as kustd1514b). The loci kustd1514 and KSMBR1_1301 share almost 100% identity across the whole length of the sequence (Fig. 3B). In contrast, kustd1514/KSMBR1_1301, kustd1514b, and KsCSTR_09970 shared some regions with almost 100% identities and some regions with only 20–60% identities. It seems that kustd1514b is a hybrid of kustd1514 and KsCSTR_09970. A search in the NCBI nr database only found six more proteins (all in *Planctomycetes*) that were distantly related to the three proteins mentioned here and which had a homologous region of at least 50% of the query sequence (Fig. S3).

In the resequencing data of KUST in 2012 where kustd1514b was found, a unique partial sequence of the original kustd1514 (between amino acid position 155 and position 726) was found in two short and low-coverage contigs 318 and 458 (coverage 1.3 ~ 1.5, overall coverage was ~ 74) (Additional file 1). Therefore, it seems that the population that contained the original kustd1514 still existed when the microbial community was re-sequenced in 2012.

Although we did not analyze glycosylation of KsCSTR_09970, proteomic data suggested that the protein KsCSTR_09970 was similarly glycosylated as kustd1514b. In a total of 10,045 peptide spectrum matches (PSMs) obtained from a large collection of proteomic data of strain CSTR1 which were associated with 46 tryptic peptides in KsCSTR_09970 (Table S12) only one single PSM (for the peptide at position 590–624) had an 11-aa overlap with one of the eight peptides that were reported to be glycosylated [32] and that were shared between kustd1514b and KsCSTR_09970 (Fig. S4). This is in agreement with the observation by van Teeseling et al. [32] who found almost no non-glycosylated variants of the detected peptides in kustd1514b.

## Discussion

The analysis of the complete genome of “*Ca.* Kuenenia stuttgartiensis” strain CSTR1 provides evidence for strong genome structure conservation in bacteria of the species “*Ca.* Kuenenia stuttgartiensis”. By comparing the CSTR1 genome with the genome of strain MBR1, we found that three regions (0.6–1.4 Mbp in length) are conserved in both their orientations and locations (Fig. 1). Apart from the conserved regions, we observed considerable rearrangements, and almost always found transposase genes at the flanks of the rearranged genomics blocks, which was consistent with previous findings showing evidence of high transposase activities in “*Ca.* Kuenenia” strains [15, 33]. Abundant transposases were also detected in the proteome of “*Ca.* Brocadia” [34], suggesting similar transposase activities in “*Ca.* Brocadia”. Since transposase activities facilitate genetic exchanges and mutations which could be beneficial to the microorganisms [28], the anammox bacteria in the natural environment may require such activities to adapt themselves to changing environment.

Comparison of the longest CRISPR locus in the genomes of strain KUST, strain MBR1, and strain CSTR1 suggested that strain KUST might have differentiated from the common ancestor of the three strains earlier than when strain MBR1 and strain CSTR1 differentiated from each other. This is in contrast to phylogenetic analyses with rRNA gene sequences, ANI, and concatenated conserved proteins that suggest that CSTR1 and KUST are more similar to each other than both are to strain MBR1. Such discrepancy can be explained by a horizontal gene transfer event of the whole CRISPR locus, meaning that the CRISPR locus does not necessarily represent the immune history in the strain. A second explanation could be that strain MBR1 evolved faster than the other two strains due to faster growth or higher mutation rates.

The results from proteome analysis in strain CSTR1 were mostly in agreement with previous findings in terms of the key enzymes in the anammox metabolism (except the annotated cytochrome *cd*_1_-type nitrite reductase, *nirS*) and other abundant proteins. A number of differences to previous studies were noticed including the division ring protein and an ammonium sensor protein. The absence of the described division ring protein KsCSTR_10780 (kustd1438, KSMBR1_1135) [31] was puzzling as the culture was at the highest growth rate for anammox bacteria (0.33 d^− 1^) and the protein is a huge protein (3690 aa) with as many as 65 tryptic peptides that are suitable for mass spectrometry. Possible reasons for the absence include too low expression levels, or unknown reasons that prevent tryptic digestion of the protein, or unknown protein modifications that prevent peptide identification. Similar to the division ring protein, a previously characterized ammonium sensor protein KsCSTR_21800 (kuste3690, KSMBR1_3866) [30] was also absent in the proteome.

The S-layer protein sequence in strain CSTR1 (KsCSTR_09970) differed significantly from that in strain KUST (kustd1514) and in KUST2012 (kustd1514b). KsCSTR_09970 is similarly glycosylated as its homolog kustd1514b. Since KsCSTR_09970 in our analyses and also kustd1514 and kustd1514b [29, 32] were all highly expressed, they appear to fulfill the same function as an S-layer protein in “*Ca.* Kuenenia” strains despite their dissimilar sequences. These suggested that the anammox bacteria may employ diverse proteins to perform the same functions.

Due to the inconsistent expression level of the annotated cytochrome *cd*_1_-type nitrite reductase (NirS, kuste4136), it was hypothesized that two putative hydroxylamine oxidoreductase genes (kustc0458 and kuste4574) may take the role of producing nitric oxide from nitrite in “*Ca.* Kuenenia” [27]. Consistent with the hypothesis, in our study we did not detect KsCSTR_33370 (analog of kuste4136) in the proteome of strain CSTR1 at high growth rates, but found KsCSTR_49490 (analog of kustc0458) and KsCSTR_29630 (kuste4574) at high expression levels. Further experiments are needed to verify whether these two candidates are representing nitrite reductase activity in growing “*Ca.* Kuenenia” strains. Interestingly, when the anammox cells are under stress of high temperature, high nitrite, or starvation, we did observe NirS in the proteome (Table S11). This suggested that the NirS in strain CSTR1 might be involved in other functions apart from nitrite reduction or in stress response.

## Conclusions

In this study, the complete genome of “*Ca.* Kuenenia stuttgartiensis” strain was sequenced and circularized. High average nucleotide identity was observed between the genome of strain CSTR1 and the genomes of “*Ca.* Kuenenia stuttgartiensis” MBR1 and KUST, while considerable differences were observed among the genomes, including rearrangements of large genomic regions, changes in CRISPR elements, and sequence shifts in the strongly expressed S-layer protein. Such differences suggested that, while “*Ca.* Kuenenia” strains showed stability of basic physiological traits, they evolved in other key aspects such as phage defense components and surface properties. Such changes may help the strain to cope with changing environments but the exact causes and consequences of the changes are not clear.

The identity of the actual nitrite reductase in “*Ca.* Kuenenia” is still unclear. We showed that the annotated cytochrome *cd*_1_-type nitrite reductase (NirS) is at least dispensable in “Ca. Kuenenia”, due to its absence in the whole proteome of strain CSTR1. The expression of this NirS under stress or starvation conditions suggested that it could be variably induced.

## Methods

### Source of the inoculum

Anammox granules were obtained from an anammox reactor treating landfill leachate in Beijing University of Technology (reactor R11 in [16]). The biomass was cultivated in anammox medium in serum bottles and became planktonic after a few transfers of granule-free liquid. The planktonic culture was later used to inoculate a 2-l semi-CSTR anammox reactor fed with 20 mM nitrite and 20 mM ammonium at an hydraulic retention time of 3 days [17].

### Cultivation and DNA extraction

Reactor effluent from the 2-l semi-CSTR anammox reactor was collected in April 2017. The abundance of the anammox population in the reactor at the time of collection was estimated to be around 87% by fluorescence in-situ hybridization. Cells were pelleted from 2 l of the reactor effluent by centrifugation at 5000 g, 4 °C for 15 min. The obtained wet cells (452 mg) were sent with ice packs to Max Planck-Genome-Centre Cologne for DNA extraction, library construction, and sequencing.

### Sequencing and genome assembly

A genomic DNA library of 20–30 kbp insert size was prepared with DNA extracted from the wet cells as described above, and sequenced on a PacBio RS II sequencer using one SMRT cell with the P6-C4 reagent. In total, 79,588 reads were generated, amounting to a total data size of 875 Mbp. Read length N50 was 15,927 bp, and subread length N50 was 9475 bp. Genome assembling was done using SMRT Analysis v2.3.0. The assembly protocol HGAP.2 was used with default settings.

A total of 183 contigs were obtained after the above-mentioned automatic assembly pipeline, of which the longest contig had a length of 4,341,910 bp and an average coverage of 93 while the other contigs had a maximal length of 54,306 bp and a maximal coverage of 33 (Table S2). Initial BLAST search of the longest contig showed homology to “*Ca.* Kuenenia”. We then took the longest contig and circularized the contig using Circlator [35] (github.com/sanger-pathogens/circlator). The circularized contig (4,334,644 bp) was then used as a reference genome to which the original reads were mapped using the resequencing protocol of SMRT Analysis v2.3.0 in order to correct errors. Average coverage was 104 and was consistent along the entire contig (Fig. S5). The finalized circular contig had a size of 4,334,932 bp, and contained a single copy of 16S rRNA gene sequence. This contig was considered to be the complete genome of the anammox population in the reactor.

Assembly of the resequencing data of strain KUST in 2012 [12] was done based on the IonTorrent reads downloaded from ebi.ac.uk (reads archive number: ERR342261) using SPAdes v3.13.0 under default settings (Additional file 1).

### Sequence analyses

Annotation of the genome of strain CSTR1 was done through the automatic annotation pipeline provided by MicroScope [20] with manual curation. Potentially missed protein coding sequences were added after comparison with the genome of strain MBR1 and KUST. Coding sequences were then refined by applying the maximum allowed sequence overlap of 60 bp [36]: if two coding sequences shared at least 60 bp overlap and the overlap was not caused by misprediction of start codons, the coding sequence that was shorter was removed unless it encoded a protein of which the function was predicted or it was found in the proteome of strain CSTR1. For pangenome and core-genome analysis, the three anammox genomes were re-annotated in Prokka pipeline [19] using Prodigal [18] as the coding sequence prediction tool. Pangenome analysis was performed in KBase [37] using default settings.

CRISPR elements were identified using CRISPRcasFinder on the online server (crisprcas.i2bc.paris-saclay.fr) [38].

Correlation of tetranucleotide signatures between genomes was calculated using the program Tetra v1.02 [23]. Average nucleotide identity (ANI) between genomes was calculated on the online server (ezbiocloud.net/tools/ani) with default settings which uses the OrthoANIu algorithm based on USEARCH [21, 22]. Concatenated conserved protein analysis was done as described previously using a set of 50 conserved genes including ribosomal proteins, DNA-directed RNA polymerase subunits, and other proteins [9]. All 50 chosen genes are present in full length in genomes of “*Ca.* Kuenenia stuttgartiensis” strains.

Alignment of genomic blocks and visualization was done using Mauve v2.3.1 with Progressive Mauve algorithm and a seed weight of 15 [39]. Genome rearrangement steps were calculated using the online server grimm.ucsd.edu/GRIMM based on GRIMM v2.01 [40]. Alignment of gene and protein sequences was done using MEGA7 [41] with settings noted in the figure legends.

### Proteomics analyses

Samples for proteomics analysis were taken from the laboratory semi-CSTR reactor of a liquid volume of 1 l. The reactor was running at an HRT of 3 days with inflowing nitrite and ammonium concentrations of 60 mM. Three replicates of samples were taken on the same day from the reactor. Supernatant was removed after centrifugation at 5000 g, 16 °C for 10 min, and cell pellets were resuspended in ammonium bicarbonate buffer (50 mM). Three cycles of freeze/thaw (− 80 °C / + 40 °C) were used to disrupt cells. Protein extracts were treated sequentially with 62.5 mM dithiothreitol and 128 mM 2-iodacetamide before digestion with reductively methylated trypsin (Promega) overnight.

Digested peptides were desalted using C_18_ ziptips (Millipore, Merck), vacuum dried, and reconstituted in 0.1% formic acid before nano-liquid chromatography–mass spectrometry (nano-LC-MS) analysis. Protein from around 3 × 10^6^ cells was injected in each nano-LC-MS/MS run. Nano-LC-MS analysis was done on a nano-LC system (Dionex Ultimate 3000RSLC, Thermo Scientific) equipped with an Orbitrap Fusion Tribrid mass spectrometer (Thermo Scientific). Peptides were separated on an Acclaim PepMap 100 C_18_ column (100 Å pore size, 3 μm particle size, 75 μm × 250 mm, Thermo Scientific) at a flow rate of 300 nL min^− 1^ and a column oven temperature of 35 °C. Mobile phase was mixed from solution A (0.1% formic acid in water) and solution B (0.08% formic acid in 20% water + 80% acetonitrile). Pump gradient was as follows: solution B (%) was 4% for 1 min, ramped up to 10% from 1 min to 5 min, slowly ramped up to 35% from 5 min to 100 min, ramped up to 55% from 100 min to 120 min, quickly ramped up to 90% from 120 min to 130 min, held at 90% from 130 min to 135 min, went down to 4% from 135 min to 137 min, and held at 4% from 137 min to 145 min. Peptides were ionized in TriVersa NanoMate, Advion electrospray ion source. Mass spectrometry analysis was performed in positive mode. Both MS1 and MS2 scans were performed on the Orbitrap mass analyzer with a MS1 resolution of 120,000 and a MS2 resolution of 60,000. Only ions with a charge state between 2 and 4 were selected for fragmentation.

Acquired raw data were analyzed using Proteome Discoverer (v2.4, Thermo Fisher Scientific). MS2 spectra were searched using SequestHT against a fasta file containing all protein sequences in the genome of “*Ca.* Kuenenia stuttgartiensis” strain CSTR1 (NCBI accession number CP049055). Mass tolerance for precursor ion mass and fragment ion mass was 3 ppm and 0.5 Da, respectively. Maximal two missed cleavage sites were allowed. Oxidation on methionine residues was set as a dynamic modification and carbamidomethylation on cysteine residues was set as a fixed modification. Protein and peptides abundance values were calculated by intensity-based label free quantification using the Minora node implemented in Proteome Discoverer. Rankings in abundance were calculated by comparing all protein abundances within one sample and ranking them according to their MS1 intensity value. The proteomics data have been deposited to the ProteomeXchange Consortium via the PRIDE partner repository with the dataset identifier PXD018553.

## Supplementary Information

**Additional file 1** Fig. S1. Cumulative GC skew along the genomes of “*Ca.* Kuenenia stuttgartiensis” strains. Fig. S2. Venn diagram showing the core genome and the genes specific in “*Ca.* Kuenenia stuttgartiensis” strains. Fig. S3. Unrooted phylogenetic trees of ten S-layer homologous gene constructed based on the Neighbor-Joining method. Fig. S4. Amino acid sequence of the S-layer protein of strain CSTR1 (KsCSTR_09970). Fig. S5. Coverage of SMRT sequencing reads along the genome of “*Ca.* Kuenenia stuttgartiensis” strain CSTR1. Table S1. Reported genome assemblies of anammox bacteria up to this study (as of 02.02.2020). Table S2. List of the longest 25 contigs obtained by the automatic assembling pipeline in the PacBio SMRT Analysis software package. Table S3. Hypothetical sequential rearrangement events from genome CSTR1 to genome MBR1b as calculated by GRIMM. Table S4. List of the 20 locally collinear blocks (LCBs) of the genome of “*Ca.* Kuenenia stuttgartiensis” strain CSTR1 after Mauve alignment with MBR1b. Table S5. CRISPR elements in the anammox genomes KUST, MBR1 and CSTR1. Table S6. Comparison of the large CRISPR locus near the type I-B CRISPR-Cas cluster in the three studied anammox genomes. Table S7. Protein-coding genes of “*Ca.* Kuenenia stuttgartiensis” strain CSTR1 and their abundance in the proteome. Table S8. Transposase genes and their classification in the anammox genomes KUST, MBR1 and CSTR1. Table S9: List of transposase genes and their classification in the anammox genomes KUST, MBR1 and CSTR1. Table S10. Abundances of peptides from two highly similar hydrazine dehydrogenases KsCSTR_46980 and KsCSTR_11820 in the proteome of “*Ca.* Kuenenia stuttgartiensis” strain CSTR1. Table S11. Detection of three nitrite reductase gene candidates in the proteome of “*Ca.* Kuenenia stuttgartiensis” strain CSTR1 over time. Table S12. List of peptides detected in the S-layer protein KsCSTR_09970 in a series of “*Ca.* Kuenenia stuttgartiensis” strain CSTR1 samples. Additional file 1. Genome assembly from the IonTorrent-based resequencing of “*Ca.* Kuenenia stuttgartiensis” strain KUST in 2012.

## Data Availability

The genome sequencing data of “*Ca.* Kuenenia stuttgartiensis” strain CSTR1 have been submitted to the National Center for Biotechnology Information (NCBI) in the BioProject PRJNA603163 (BioSample: SAMN13921532; reads archive number: SRR11213620; genome accession number CP049055). The mass spectrometry proteomics data have been deposited to the ProteomeXchange Consortium via the PRIDE partner repository with the dataset identifier PXD018553. The IonTorrent reads of the resequencing project for “Ca. Kuenenia stuttgartiensis” strain KUST in 2012 was downloaded from the European Molecular Biology Laboratory’s European Bioinformatics Institute (EMBL-EBI) under the reads archive number ERR342261.

## Abbreviations

Anammox: Anaerobic ammonium oxidation
SMRT sequencing: Single-molecule real-time sequencing
CSTR: Continuous stirred-tank reactor
CRISPR: Clustered regularly interspaced short palindromic repeats
bp: Base pair
ori: Origin of replication
ANI: Average nucleotide identity
LCB: Locally collinear block
HRT: Hydraulic retention time
PSM: Peptide spectrum match
nano-LC-MS: Nano-liquid chromatography–mass spectrometry
CDS: Coding sequence
aa: Amino acids.

## References

[1] Wang S, Zhu G, Zhuang L, Li Y, Liu L, Lavik G, Berg M, Liu S, Long X-E, Guo J (2020). Anaerobic ammonium oxidation is a major N-sink in aquifer systems around the world. ISME J.

[2] McCarty PL (2018). What is the best biological process for nitrogen removal: when and why?. Environ Sci Technol.

[3] Zhang L, Okabe S (2020). Ecological niche differentiation among anammox bacteria. Water Res.

[4] Zheng B, Zhang L, Guo J, Zhang S, Yang A, Peng Y (2016). Suspended sludge and biofilm shaped different anammox communities in two pilot-scale one-stage anammox reactors. Bioresour Technol.

[5] Bhattacharjee AS, Wu S, Lawson CE, Jetten MSM, Kapoor V, Domingo JWS, McMahon KD, Noguera DR, Goel R (2017). Whole community metagenomics in two different anammox configurations: process performance and community structure. Environ Sci Technol.

[6] Hu B-L, Zheng P, Tang C-J, Chen J-W, van der Biezen E, Zhang L, Ni B-J, Jetten MSM, Yan J, Yu H-Q (2010). Identification and quantification of anammox bacteria in eight nitrogen removal reactors. Water Res.

[7] Strous M, Fuerst JA, Kramer EHM, Logemann S, Muyzer G, van de Pas-Schoonen KT, Webb R, Kuenen JG, Jetten MSM (1999). Missing lithotroph identified as new planctomycete. Nature.

[8] Peeters SH, van Niftrik L (2019). Trending topics and open questions in anaerobic ammonium oxidation. Curr Opin Chem Biol.

[9] Strous M, Pelletier E, Mangenot S, Rattei T, Lehner A, Taylor MW, Horn M, Daims H, Bartol-Mavel D, Wincker P (2006). Deciphering the evolution and metabolism of an anammox bacterium from a community genome. Nature.

[10] Dietl A, Ferousi C, Maalcke WJ, Menzel A, de Vries S, Keltjens JT, Jetten MSM, Kartal B, Barends TRM (2015). The inner workings of the hydrazine synthase multiprotein complex. Nature.

[11] Maalcke WJ, Reimann J, de Vries S, Butt JN, Dietl A, Kip N, Mersdorf U, Barends TRM, Jetten MSM, Keltjens JT (2016). Characterization of anammox hydrazine dehydrogenase, a key N_2_-producing enzyme in the global nitrogen cycle. J Biol Chem.

[12] van Teeseling MCF, de Almeida NM, Klingl A, Speth DR (2014). Op den camp HJM, Rachel R, Jetten MSM, van Niftrik L: a new addition to the cell plan of anammox bacteria: “*Candidatus* Kuenenia stuttgartiensis” has a protein surface layer as the outermost layer of the cell. J Bacteriol.

[13] Kartal B, Maalcke WJ, de Almeida NM, Cirpus I, Gloerich J, Geerts W, HJM O d C, Harhangi HR, Janssen-Megens EM, Francoijs K-J (2011). Molecular mechanism of anaerobic ammonium oxidation. Nature.

[14] de Almeida NM, Wessels HJCT, de Graaf RM, Ferousi C, Jetten MSM, Keltjens JT, Kartal B. Membrane-bound electron transport systems of an anammox bacterium: a complexome analysis. Biochim Biophys Acta. 2016;1857:1694–1704. 10.1016/j.bbabio.2016.07.006 27461995

[15] Frank J, Lücker S, Vossen RHAM, Jetten MSM, Hall RJ (2018). Op den camp HJM, Anvar SY: resolving the complete genome of *Kuenenia stuttgartiensis* from a membrane bioreactor enrichment using single-molecule real-time sequencing. Sci Rep.

[16] Ding C, Adrian L, Peng Y, He J (2020). 16S rRNA gene-based primer pair showed high specificity and quantification accuracy in detecting freshwater Brocadiales anammox bacteria. FEMS Microbiol Ecol.

[17] Ding C, Enyi FO, Adrian L (2018). Anaerobic ammonium oxidation (anammox) with planktonic cells in a redox-stable semicontinuous stirred-tank reactor. Environ Sci Technol.

[18] Hyatt D, Chen G-L, LoCascio P, Land M, Larimer F, Hauser L (2010). Prodigal: prokaryotic gene recognition and translation initiation site identification. BMC Bioinform.

[19] Seemann T (2014). Prokka: rapid prokaryotic genome annotation. Bioinformatics.

[20] Vallenet D, Calteau A, Dubois M, Amours P, Bazin A, Beuvin M, Burlot L, Bussell X, Fouteau S, Gautreau G (2019). MicroScope: an integrated platform for the annotation and exploration of microbial gene functions through genomic, pangenomic and metabolic comparative analysis. Nucleic Acids Res.

[21] Yoon S-H (2017). Ha S-m, Lim J, kwon S, Chun J: a large-scale evaluation of algorithms to calculate average nucleotide identity. Antonie Van Leeuwenhoek.

[22] Lee I, Ouk Kim Y, Park S-C, Chun J (2016). OrthoANI: an improved algorithm and software for calculating average nucleotide identity. Int J Syst Evol Microbiol.

[23] Teeling H, Waldmann J, Lombardot T, Bauer M, Glockner F (2004). TETRA: a web-service and a stand-alone program for the analysis and comparison of tetranucleotide usage patterns in DNA sequences. BMC Bioinform.

[24] Barrangou R, Marraffini LA (2014). CRISPR-Cas systems: prokaryotes upgrade to adaptive immunity. Mol Cell.

[25] Alkhnbashi OS, Shah SA, Garrett RA, Saunders SJ, Costa F, Backofen R (2016). Characterizing leader sequences of CRISPR loci. Bioinformatics.

[26] Shoji S, Dambacher CM, Shajani Z, Williamson JR, Schultz PG (2011). Systematic chromosomal deletion of bacterial ribosomal protein genes. J Mol Biol.

[27] Kartal B, de Almeida NM, Maalcke WJ, HJM O d C, MSM J, Keltjens JT (2013). How to make a living from anaerobic ammonium oxidation. FEMS Microbiol Rev.

[28] Aziz RK, Breitbart M, Edwards RA (2010). Transposases are the most abundant, most ubiquitous genes in nature. Nucleic Acids Res.

[29] Neumann S, Wessels HJCT, Rijpstra WIC, Sinninghe Damsté JS, Kartal B, Jetten MSM, van Niftrik L (2014). Isolation and characterization of a prokaryotic cell organelle from the anammox bacterium *Kuenenia stuttgartiensis*. Mol Microbiol.

[30] Pflüger T, Hernández CF, Lewe P, Frank F, Mertens H, Svergun D, Baumstark MW, Lunin VY, Jetten MSM, Andrade SLA (2018). Signaling ammonium across membranes through an ammonium sensor histidine kinase. Nat Commun.

[31] Lv N, Geerts WJC, EGv D, Humbel BM, Webb RI, Harhangi HR, HJMOd C, Fuerst JA, Verkleij AJ, MSM J (2009). Cell division ring, a new cell division protein and vertical inheritance of a bacterial organelle in anammox planctomycetes. Mol Microbiol.

[32] van Teeseling MCF, Maresch D, Rath CB, Figl R, Altmann F, Jetten MSM, Messner P, Schäffer C, van Niftrik L. The s-layer protein of the anammox bacterium *Kuenenia stuttgartiensis* is heavily o-glycosylated. Front Microbiol. 2016;7:1721. 10.3389/fmicb.2016.01721 PMC5088730 27847504

[33] Speth DR, Hu B, Bosch N, Keltjens JT, Stunnenberg HG, Jetten MSM (2012). Comparative genomics of two independently enriched “*Candidatus* Kuenenia stuttgartiensis” anammox bacteria. Front Microbiol.

[34] Lin X, Wang Y, Ma X, Yan Y, Wu M, Bond PL, Guo J (2018). Evidence of differential adaptation to decreased temperature by anammox bacteria. Environ Microbiol.

[35] Hunt M, Silva ND, Otto TD, Parkhill J, Keane JA, Harris SR (2015). Circlator: automated circularization of genome assemblies using long sequencing reads. Genome Biol.

[36] Pallejà A, Harrington ED, Bork P (2008). Large gene overlaps in prokaryotic genomes: result of functional constraints or mispredictions?. BMC Genomics.

[37] Arkin AP, Cottingham RW, Henry CS, Harris NL, Stevens RL, Maslov S, Dehal P, Ware D, Perez F, Canon S (2018). KBase: the United States Department of Energy systems biology knowledgebase. Nat Biotechnol.

[38] Couvin D, Bernheim A, Toffano-Nioche C, Touchon M, Michalik J, Néron B, Rocha EPC, Vergnaud G, Gautheret D, Pourcel C (2018). CRISPRCasFinder, an update of CRISRFinder, includes a portable version, enhanced performance and integrates search for Cas proteins. Nucleic Acids Res.

[39] Darling ACE, Mau B (2010). Perna NT: progressiveMauve: multiple genome alignment with gene gain, loss and rearrangement. PLoS One.

[40] Tesler G (2002). GRIMM: genome rearrangements web server. Bioinformatics.

[41] Kumar S, Stecher G, Tamura K. MEGA7: Molecular Evolutionary Genetics Analysis version 7.0 for bigger datasets. Mol Biol Evol. 2016;33:1870–4. 10.1093/molbev/msw054 PMC8210823 27004904

