# Supplementary material for: Comparative genomics in “Candidatus Kuenenia stuttgartiensis” reveal high genomic plasticity in the overall genome structure, CRISPR loci and surface proteins
Source: BMC Genomics. 2020 Dec 1;21:851. doi: 10.1186/s12864-020-07242-1 (PMC7709395; doi:10.1186/s12864-020-07242-1)
Supplement: Supplementary file 1 — Additional file 1 Fig. S1. Cumulative GC skew along the genomes of “Ca. Kuenenia stuttgartiensis” strains. Fig. S2. Venn diagram showing the core genome and the genes specific in “Ca. Kuenenia stuttgartiensis” strains. Fig. S3. Unrooted phylogenetic trees of ten S-layer homologous gene constructed based on the Neighbor-Joining method. Fig. S4. Amino acid sequence of the S-layer protein of strain CSTR1 (KsCSTR_09970). Fig. S5. Coverage of SMRT sequencing reads along the genome of “Ca. Kuenenia stuttgartiensis” strain CSTR1. Table S1. Reported genome assemblies of anammox bacteria up to this study (as of 02.02.2020). Table S2. List of the longest 25 contigs obtained by the automatic assembling pipeline in the PacBio SMRT Analysis software package. Table S3. Hypothetical sequential rearrangement events from genome CSTR1 to genome MBR1b as calculated by GRIMM. Table S4. List of the 20 locally collinear blocks (LCBs) of the genome of “Ca. Kuenenia stuttgartiensis” strain CSTR1 after Mauve alignment with MBR1b. Table S5. CRISPR elements in the anammox genomes KUST, MBR1 and CSTR1. Table S6. Comparison of the large CRISPR locus near the type I-B CRISPR-Cas cluster in the three studied anammox genomes. Table S7. Protein-coding genes of “Ca. Kuenenia stuttgartiensis” strain CSTR1 and their abundance in the proteome. Table S8. Transposase genes and their classification in the anammox genomes KUST, MBR1 and CSTR1. Table S9: List of transposase genes and their classification in the anammox genomes KUST, MBR1 and CSTR1. Table S10. Abundances of peptides from two highly similar hydrazine dehydrogenases KsCSTR_46980 and KsCSTR_11820 in the proteome of “Ca. Kuenenia stuttgartiensis” strain CSTR1. Table S11. Detection of three nitrite reductase gene candidates in the proteome of “Ca. Kuenenia stuttgartiensis” strain CSTR1 over time. Table S12. List of peptides detected in the S-layer protein KsCSTR_09970 in a series of “Ca. Kuenenia stuttgartiensis” strain CSTR1 samples. Additional [file 12864_2020_7242_MOESM1_ESM.zip › SupplementaryMaterial.docx]

Supplementary information

**Comparative genomics in “*Candidatus* Kuenenia stuttgartiensis” reveal high genomic plasticity in the overall genome structure, CRISPR loci and surface proteins**

Chang Ding^1,*^, Lorenz Adrian^1,2^

*To whom correspondence should be addressed: Chang Ding, Helmholtz Centre for Environmental Research – UFZ, Environmental Biotechnology, Permoserstraße 15, 04318 Leipzig, Germany, Tel.: +49 (0) 341 235 1412, Fax: +49 (0) 341 235 1443, E-Mail: chang.ding@ufz.de

Index:

[Figure S1: Cumulative GC skew along the genomes of “*Ca.* Kuenenia stuttgartiensis” strains. 2](#_Toc56175442)

[Figure S2: Venn diagram showing the core genome and the genes specific in “*Ca.* Kuenenia stuttgartiensis” strains. 3](#_Toc56175443)

[Figure S3. Unrooted phylogenetic trees of ten S-layer homologous genes constructed based on the Neighbor-Joining method. (A) Tree constructed with gene sequences. (B) Tree constructed with protein sequences. Trees are drawn to scale, with branch lengths measured in the number of substitutions per site. Bootstrap values (500 replicates) at the nodes were all > 0.98 and were not shown. 4](#_Toc56175444)

[Figure S4: Amino acid sequence of the S-layer protein of strain CSTR1 (KsCSTR_09970). 5](#_Toc56175445)

[Figure S5: Coverage of SMRT sequencing reads along the genome of “*Ca.* Kuenenia stuttgartiensis” strain CSTR1. Window size: 50 kbp. Step size: 10 kbp. 6](#_Toc56175446)

[Table S1: Reported genome assemblies of anammox bacteria up to this study (as of 02.02.2020) 8](#_Toc56175449)

[Table S2: List of the longest 25 contigs obtained by the automatic assembling pipeline in the PacBio SMRT Analysis software package. 9](#_Toc56175450)

[Table S3: Hypothetical sequential rearrangement events from genome CSTR1 to genome MBR1b as calculated by GRIMM [4] 10](#_Toc56175451)

[Table S4: List of the 20 locally collinear blocks (LCBs) of the genome of “*Ca.* Kuenenia stuttgartiensis” strain CSTR1 after Mauve alignment with MBR1b 11](#_Toc56175452)

[Table S5: CRISPR elements in the anammox genomes KUST, MBR1 and CSTR1. 12](#_Toc56175453)

[Table S6: Comparison of the large CRISPR locus near the type I-B CRISPR-Cas cluster in the three studied anammox genomes. 13](#_Toc56175454)

[Table S7: Protein-coding genes of “*Ca.* Kuenenia stuttgartiensis” strain CSTR1 and their abundance in the proteome. Homologous genes in the genome of strain MBR1 and KUST (including six-frame translation) are listed. 14](#_Toc56175455)

[Table S8: Transposase genes and their classification in the anammox genomes KUST, MBR1 and CSTR1. 15](#_Toc56175456)

[Table S9: List of transposase genes and their classification in the anammox genomes KUST, MBR1 and CSTR1. 17](#_Toc56175457)

[Table S10: Abundances of peptides from two highly similar hydrazine dehydrogenases KsCSTR_46980 and KsCSTR_11820 in the proteome of “*Ca.* Kuenenia stuttgartiensis” strain CSTR1. 18](#_Toc56175458)

[Table S11: Detection of three nitrite reductase gene candidates in the proteome of “*Ca.* Kuenenia stuttgartiensis” strain CSTR1 over time. 19](#_Toc56175459)

[Table S12: List of peptides detected in the S-layer protein KsCSTR_09970 in a series of “*Ca.* Kuenenia stuttgartiensis” strain CSTR1 samples. 20](#_Toc56175460)


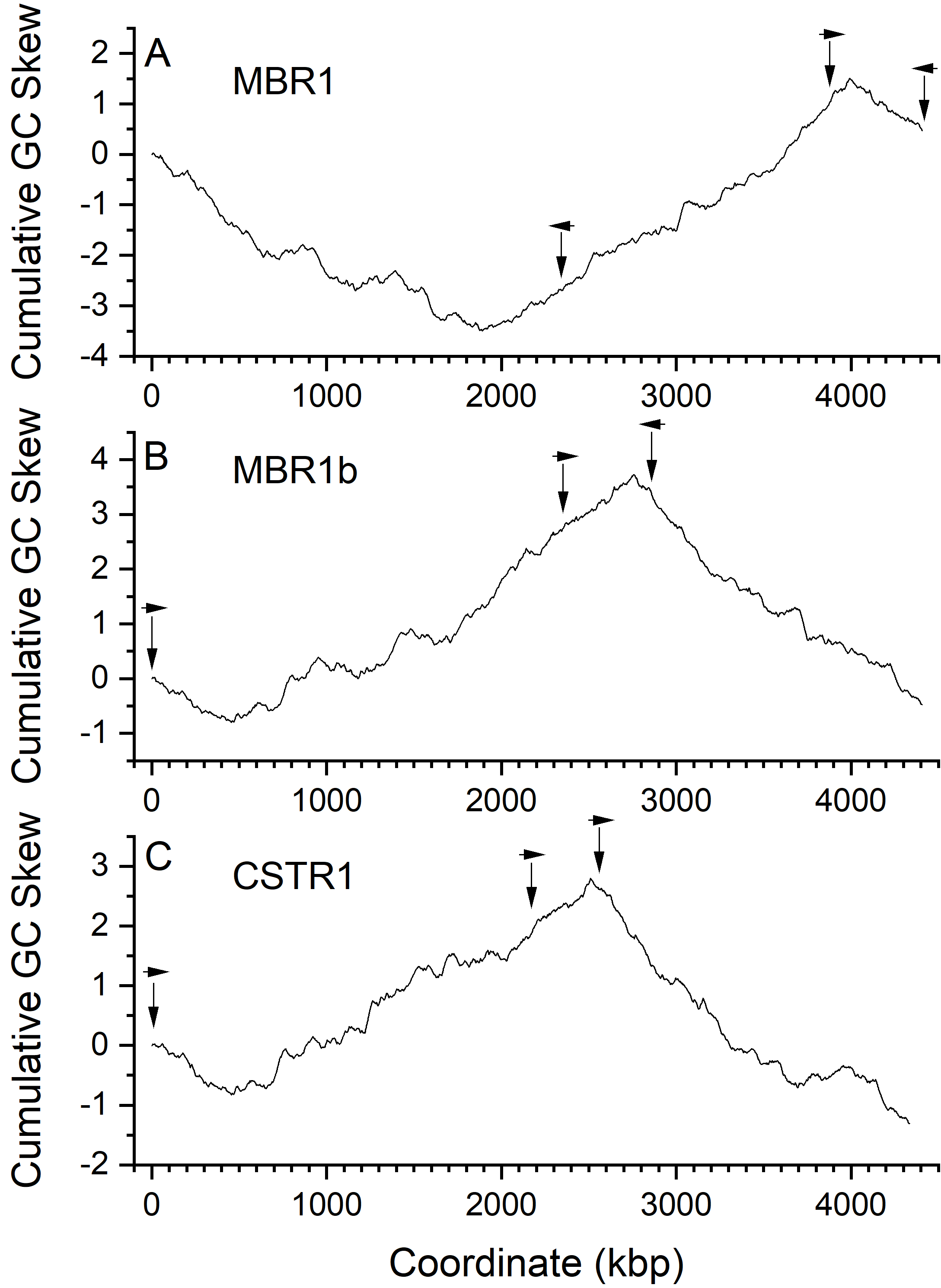


Figure S1: Cumulative GC skew along the genomes of “*Ca.* Kuenenia stuttgartiensis” strains.

Window size: 2,000 bp. step size: 1,000 bp. To avoid the dependence on the window size w and chromosome length c, the skew values are multiplied by w/c. Vertical arrows indicate locations of *dnaA* genes, and horizontal arrows indicate orientations of the *dnaA* genes.


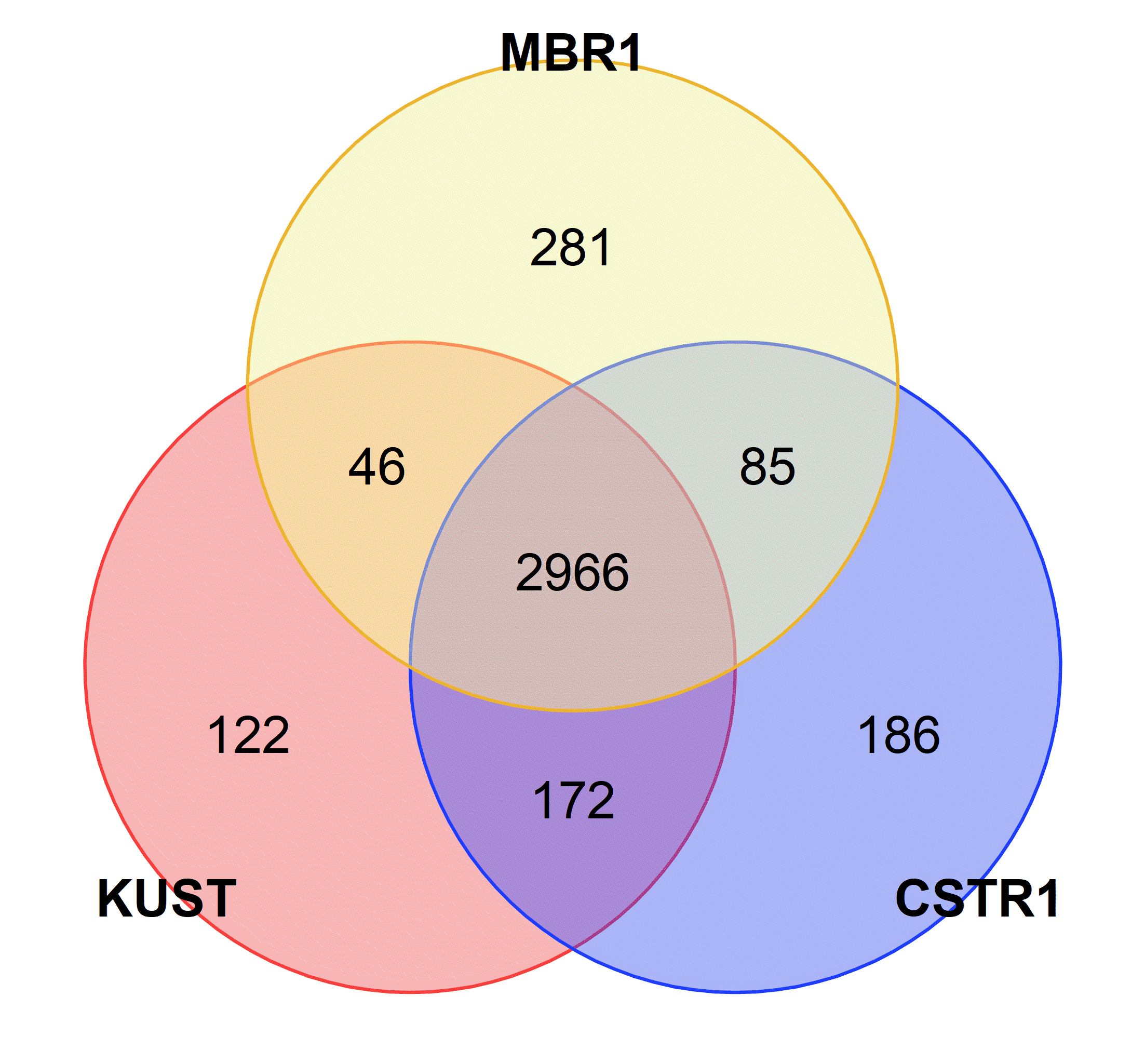


Figure S2: Venn diagram showing the core genome and the genes specific in “*Ca.* Kuenenia stuttgartiensis” strains.


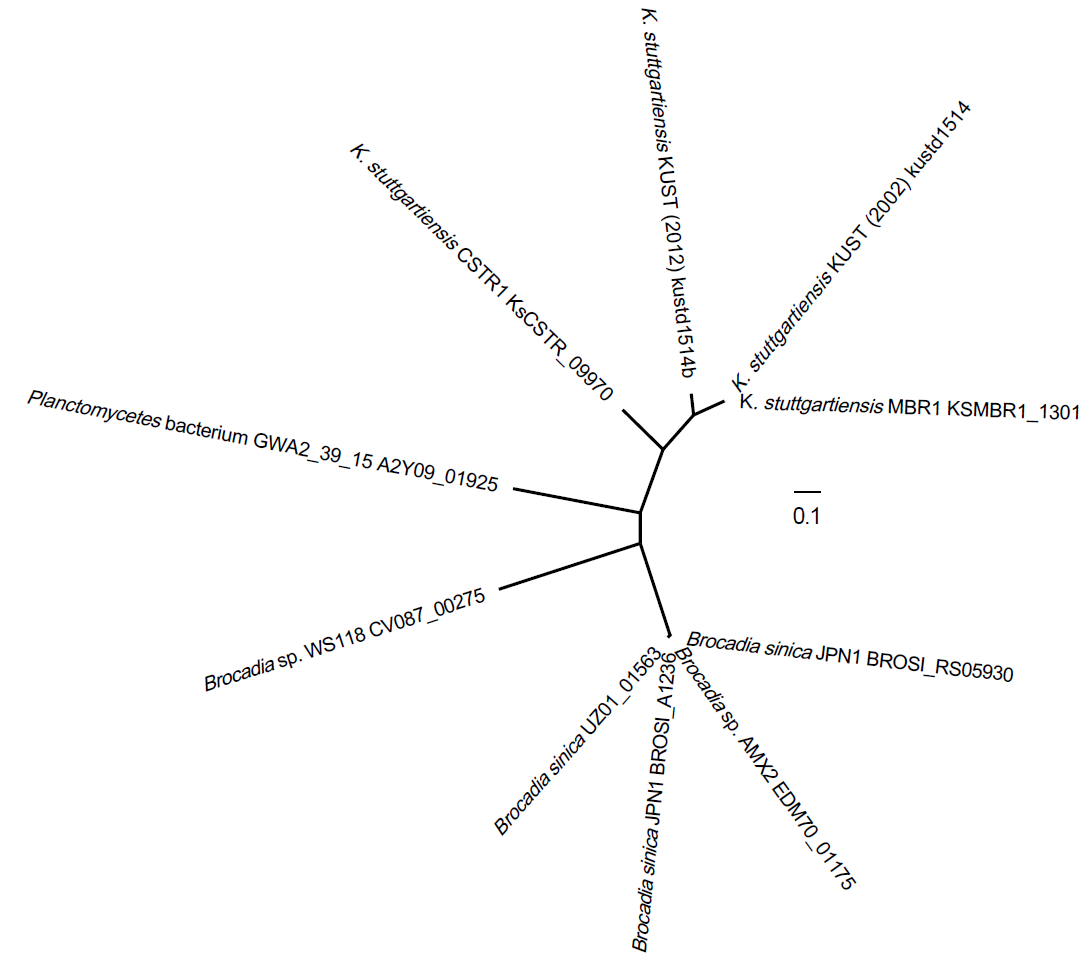


A


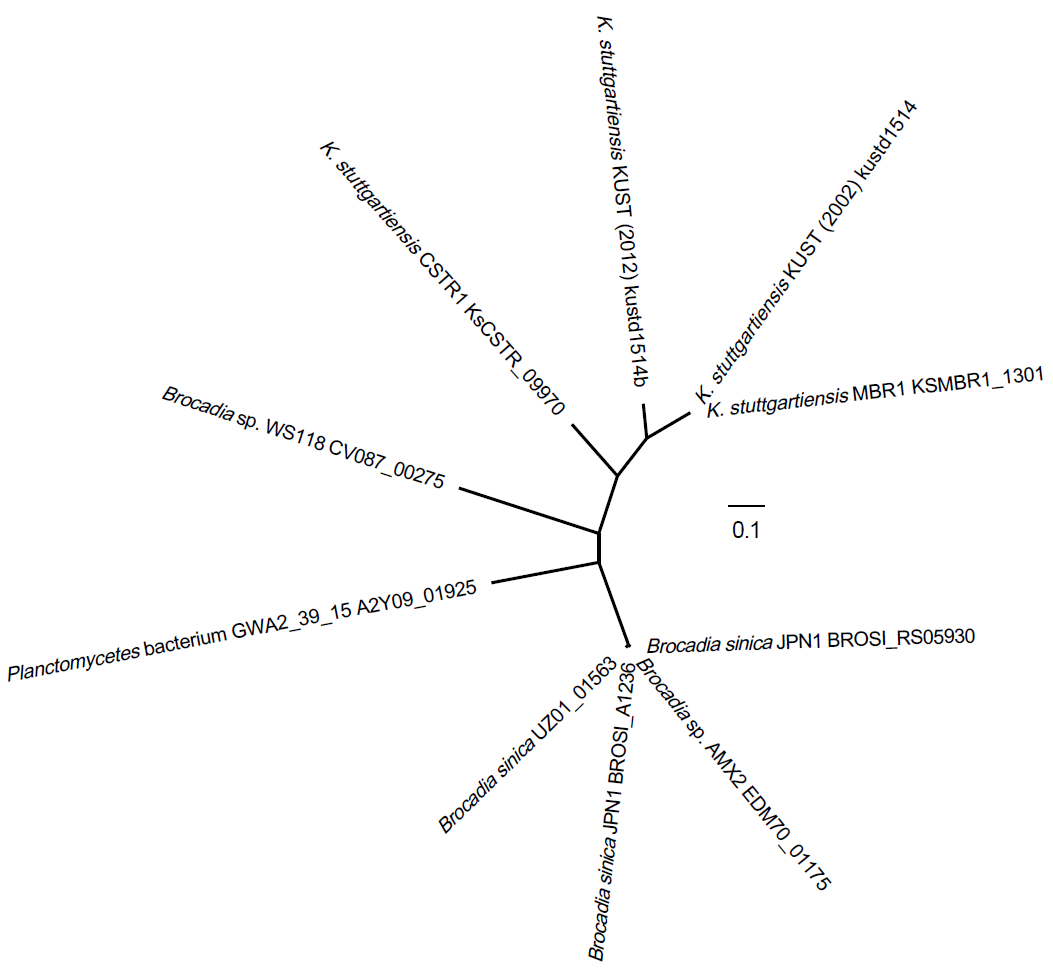


B

Figure S3. Unrooted phylogenetic trees of ten S-layer homologous genes constructed based on the Neighbor-Joining method. (A) Tree constructed with gene sequences. (B) Tree constructed with protein sequences. Trees are drawn to scale, with branch lengths measured in the number of substitutions per site. Bootstrap values (500 replicates) at the nodes were all > 0.98 and were not shown.

MKRPGLNLKGNWLSMAGVLFLMLALVMGVASNAKAATGSFDRDHYLPQLGTSDYDRAWIMVEDSTATGTTISVTVQALTSGESATYTLVGGSSTAFTTHKGQTTLPHATAIGSSTGYVEDFLGSYTYPALGTGTGALKLITGGLGSSAPGNAANGTDGVLKVNSSDTLALVYSGATLDTASVSIIGANDSTIEITTASPWGTSVDTGVNVEDATANVKITVVDPNVNLNPNLKEVIGLQDGFTTGLASAGSSRVRVQVIDQDSATGDALTGATASNIILVETGKNTGSFVATGKVYGSSSTSAKSNLRLGTTTSAFANGYDGSAITLGGPTGPSVTFQILEVTADGKLAIVGGTLTAGEVGTQTLAYAANTSAGFTDFGTQTWAADKVVVGIRDTNDEVSLFGNPTSNTSAYLPSSTRIFKLIDGANYCLVKITGSVGSTTATLSNGTYGVETGGSSAGSITVTLDAFVLAGARSGDSVKVSYLDALTAATGTFGTVTSSTAFGATGETGSVAVDKTSVDINDFFAITVVDGNLNTSSTARGSVASGDWNGTTTNSRGDRLKVAGYSNSSFVIDLQHQDGSRVGTQSVRISSTDGSLIWVVPNSLSDSTYGFRDPLTPGSSSFKLGTQATSALPLIALTKGNSTSAQSTLSSANANSFVATTDAVAGTVEISPDGTHWVAVPITETGINSSTFVGTIGFDFTAARVTTNSNTANTTSIFTDFTGTSSIIFQSPMDSTSLARTIGTGSVVRISDGIYKEFREVTGVSGTTLSVTKMQNTGFYTPWKTWVQVVGNDMEPNRADTISGTQLFRIGGYFGATYRVRYNDALNADGEYASGDNLAVTADNVTFTTNTAELSVSPSGTAGLNQTIVVTLVDGDLNTSTTVAQTTYTDTSTLPIGLNEVGLGFPSGTSTGNTSAASADLAKKNGGAAKVVFASNSSSVTSTDTSNATTSNTTHFKLVETAVNSGTFKGSFNLTPSGSTDQGTTPPQLKVVSGDTVTIYYNDSPSASNENNLQNLTTVSIVTSAREGTLSLSKSEAFLSGDSVVVTLVDSDLSGNSTQNVFVTSTSGAGSITVSLAETAVSSGEFKGTFKTGASTDSTTTPKTIRSVADGVVTVTYQENSPARDVTAQVSTKNFGAVLDITDDTVALGGSAVVSLYDPESNTSIDTANIVGTVRVTSTTDSTGVILQLNETGLDTGSFLGTILVSSDSSLQNTRIQSQTGDTITATFKDSPNASGGSSVVTDTATVGEVATPTPTPTGSVTPTTTPTASPTPTVSPIPGTGSVEGFVTDAATGDGIEGATVRNQSGIYTDTTDADGFYSIANVEAGTRTFTAVALGYVPSAPTAIVVTAGGTTNLDFALVASVQGTPTTTPTVTPPPTATLVVVVSDENGPLAGATVTVDGQSGITDASGGATFTLEAGDYEVSVSATDHITSNTTVTVTPPVTIHEVTLEIKICGEPGEVEASNATVTPDILDLVKGDSEDVIVLVTGDNECPAQGVKVKRKLTSANKKKIKVTPASQKTDRTGQATFTVKAKKNKGKANVKFGVKGVKVTPKVNVSLSK

Figure S4: Amino acid sequence of the S-layer protein of strain CSTR1 (KsCSTR_09970).

Grey-shaded: sequence regions detected in the proteome of strain CSTR1 (10,045 PSMs). Red: glycosylated peptides detected in the S-layer protein kustd1514b [[1](#_ENREF_1)]. The overlapping sequence of these two groups is framed with a box.


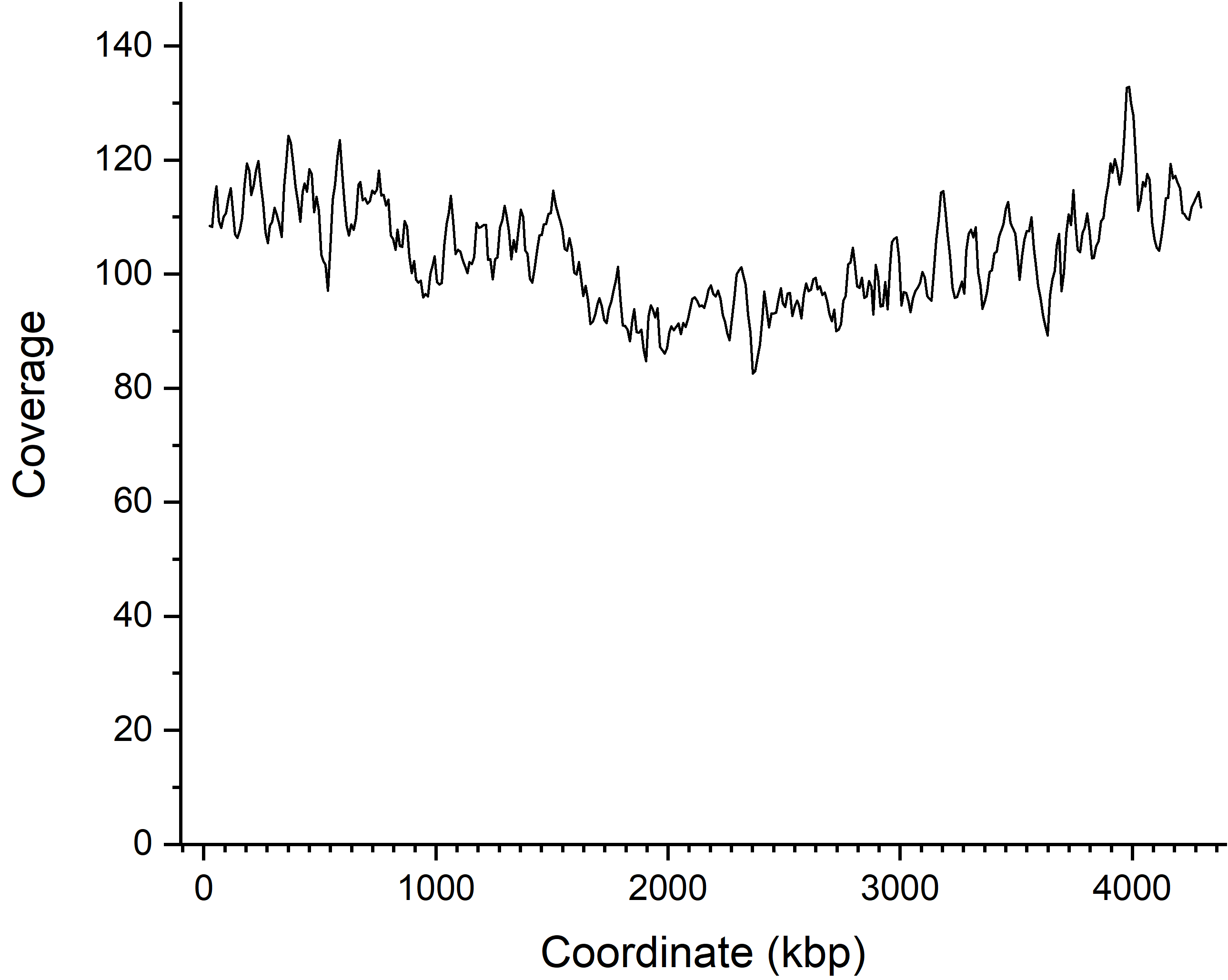


Figure S5: Coverage of SMRT sequencing reads along the genome of “*Ca.* Kuenenia stuttgartiensis” strain CSTR1. Window size: 50 kbp. Step size: 10 kbp.

Table S1: Reported genome assemblies of anammox bacteria up to this study (as of 02.02.2020)

| Organism/Name | Accession number in NCBI or JGI | #  Contigs | Size (Mb) | GC (%) | Release date |
| --- | --- | --- | --- | --- | --- |
| “*Ca.* Brocadia caroliniensis” | GCA_002009475.1 | 209 | 3.7 | 43.5 | 02.03.2017 |
| “*Ca.* Brocadia fulgida” | GCA_000987375.1 | 315 | 3.6 | 44.9 | 01.05.2015 |
| “*Ca.* Brocadia sapporoensis” (strain 40) | GCA_001753675.2 | 123 | 2.9 | 42.7 | 23.03.2017 |
| “*Ca.* Brocadia sinica” | GCA_001567345.1 | 86 | 3.9 | 42.3 | 24.02.2016 |
| “*Ca.* Brocadia sinica” JPN1 | GCA_000949635.1 | 3 | 4.1 | 42.4 | 28.02.2015 |
| “*Ca.* Brocadia” sp. AMX1 | GCA_003577215.1 | 82 | 3.8 | 42.3 | 19.09.2018 |
| “*Ca.* Brocadia” sp. AMX2 | GCA_003577195.1 | 253 | 3.5 | 45.0 | 19.09.2018 |
| “*Ca.* Brocadia” sp. AMX2 | GCA_008363395.1 | 90 | 3.9 | 42.3 | 09.09.2019 |
| “*Ca.* Brocadia” sp. AMX3 | GCA_900696475.1 | 157 | 3.8 | 40.1 | 01.03.2019 |
| “*Ca.* Brocadia” sp. BL1 | GCA_007618155.1 | 138 | 3.2 | 42.3 | 29.07.2019 |
| “*Ca.* Brocadia” sp. BROELEC01 | GCA_004282735.1 | 36 | 3.2 | 42.3 | 20.02.2019 |
| “*Ca.* Brocadia” sp. R4W10303 | GCA_002848945.1 | 168 | 3.2 | 42.3 | 28.12.2017 |
| “*Ca.* Brocadia” sp. SB37 | GCA_008933285.1 | 743 | 2.3 | 43.8 | 09.10.2019 |
| “*Ca.* Brocadia” sp. UTAMX1 | GCA_002050325.1 | 36 | 3.1 | 42.3 | 20.03.2017 |
| “*Ca.* Brocadia” sp. UTAMX2 | GCA_002050315.1 | 148 | 3.4 | 45.1 | 20.03.2017 |
| “*Ca.* Brocadia” sp. WS118 | GCA_007618135.1 | 299 | 5.0 | 43.2 | 29.07.2019 |
| “*Ca.* Jettenia asiatica” ^1^ | - | 10,343 | - | 39.4 | 2012 |
| “*Ca.* Jettenia caeni” KSU-1 | GCA_000296795.1 | 4 | 4.1 | 40.1 | 04.05.2012 |
| “*Ca.* Jettenia ecosi” J2 | GCA_005524015.1 | 223 | 3.9 | 40.1 | 20.05.2019 |
| “*Ca.* Jettenia” sp. AMX1 | GCA_008363445.1 | 104 | 3.8 | 39.8 | 09.09.2019 |
| “*Ca.* Kuenenia stuttgartiensis” AMX1 | GCA_900696675.1 | 312 | 4.0 | 40.7 | 01.03.2019 |
| “*Ca.* Kuenenia stuttgartiensis” BL10 | GCA_007618145.1 | 306 | 2.6 | 40.9 | 29.07.2019 |
| “*Ca.* Kuenenia stuttgartiensis” CH1 | GCA_000315095.1 | 1,311 | 3.8 | 40.7 | 26.11.2012 |
| “*Ca.* Kuenenia stuttgartiensis” CSTR1 | CP049055 / GCA_011066545.1 | 1 | 4.3 | 41.0 | This study |
| “*Ca.* Kuenenia stuttgartiensis” KUST | CT030148, CT573071 to CT573074 | 5 | 4.2 | 41.0 | 29.06.2009 |
| “*Ca.* Kuenenia stuttgartiensis” RU1 | GCA_000315115.1 | 2,717 | 3.8 | 41.1 | 26.11.2012 |
| “*Ca.* Kuenenia stuttgartiensis” MBR1 | LT934425 /  GCA_900232105.1 | 1 | 4.4 | 41.1 | 22.10.2017 |
| “*Ca.* Kuenenia stuttgartiensis” Ru_enrich_A3 | GCA_002632395.1 | 483 | 3.6 | 40.7 | 23.10.2017 |
| “*Ca.* Kuenenia stuttgartiensis” UBA5795 | GCA_002418245.1 | 187 | 3.7 | 40.6 | 03.10.2017 |
| “*Ca.* Scalindua brodae” | GCA_000786775.1 | 282 | 4.1 | 39.6 | 25.11.2014 |
| “*Ca.* Scalindua japonica” husup-a2 | GCA_002443295.1 | 47 | 4.8 | 38.8 | 29.09.2017 |
| “*Ca.* Scalindua profunda” ^2^ | 2017108002, 2022004002 (JGI) | 1,580 | - | 39.1 | 04.08.2015 |
| “*Ca.* Scalindua rubra” | GCA_001723765.1 | 443 | 5.2 | 37.3 | 13.09.2016 |
| “*Ca.* Scalindua rubra” Ru_enrich_A1 | GCA_002632345.1 | 100 | 4.0 | 39.7 | 23.10.2017 |
| “*Ca.* Scalindua” sp. amx | GCA_008501815.1 | 66 | 4.4 | 41.1 | 18.09.2019 |
| “*Ca.* Scalindua” sp. AMX11 | GCA_004351875.1 | 121 | 4.6 | 41.1 | 14.03.2019 |
| “*Ca.* Scalindua” sp. MohnsGC08_192 | GCA_008636105.1 | 71 | 3.0 | 38.3 | 23.09.2019 |
| “*Ca.* Scalindua” sp. SCAELEC01 | GCA_004282745.1 | 104 | 4.6 | 41.1 | 20.02.2019 |

^1^ The genome of "*Ca.* Jettenia asiatica" was reported [[2](#_ENREF_2)] but the assembly is not published.

^2^ The two genome assemblies reported for "*Ca.* Scalindua profunda" [[3](#_ENREF_3)] are unusually large (>20 Mbp) and therefore the size of the genome is not shown.

Table S2: List of the longest 25 contigs obtained by the automatic assembling pipeline in the PacBio SMRT Analysis software package.

| **Contig** | **Length (bp)** | **Coverage (×)** | **Phylogeny ^1^** |
| --- | --- | --- | --- |
| contig_1 ^2^ | 4,341,910 | 93 | “*Ca.* Kuenenia” |
| contig_2 | 54,306 | 25.42 | *Methyloversatilis* |
| contig_3 | 28,297 | 19.93 | *Methyloversatilis* |
| contig_4 | 25,636 | 24.35 | *Methyloversatilis* |
| contig_5 | 24,323 | 32.59 | *Methyloversatilis* |
| contig_6 | 23,788 | 22.88 | *Methyloversatilis* |
| contig_7 | 20,861 | 17.50 | *Methyloversatilis* |
| contig_8 | 20,363 | 20.44 | *Methyloversatilis* |
| contig_9 | 19,770 | 27.03 | *Methyloversatilis* |
| contig_10 | 18,535 | 24.69 | *Methyloversatilis* |
| contig_11 | 16,152 | 29.62 | *Pseudomonas* ^3^ |
| contig_12 | 15,874 | 25.71 | *Methyloversatilis* |
| contig_13 | 15,513 | 26.29 | *Methyloversatilis* |
| contig_14 | 15,312 | 24.37 | *Methyloversatilis* |
| contig_15 | 15,243 | 20.20 | *Methyloversatilis* |
| contig_16 | 15,177 | 24.58 | *Methyloversatilis* |
| contig_17 | 15,110 | 22.93 | *Methyloversatilis* |
| contig_18 | 15,109 | 20.14 | *Methyloversatilis* |
| contig_19 | 14,822 | 20.76 | *Methyloversatilis* |
| contig_20 | 14,668 | 26.51 | *Methyloversatilis* |
| contig_21 | 14,510 | 21.76 | *Methyloversatilis* |
| contig_22 | 14,421 | 22.28 | *Methyloversatilis* |
| contig_23 | 14,365 | 22.81 | *Methyloversatilis* |
| contig_24 | 14,301 | 34.82 | *Methyloversatilis* |
| contig_25 | 14,177 | 21.06 | *Methyloversatilis* |

^1^ Phylogeny was determined as the majority of phylogenetic affiliations suggested by the BLASTp matches of the annotated protein sequences from the contig.

^2^ contig_1 was later circularized, base-corrected, and identified as the complete genome of strain CSTR1.

^3^ contig_11 contains a majority of conjugation-related proteins from *Pseudomonas aeruginosa*.

Table S3: Hypothetical sequential rearrangement events from genome CSTR1 to genome MBR1b as calculated by GRIMM [[4](#_ENREF_4)]

| Step | Description | Locally collinear blocks (LCBs) with numbering as shown in Figure 1 | | | | | | | | | | | | | | | | | | | |
| --- | --- | --- | --- | --- | --- | --- | --- | --- | --- | --- | --- | --- | --- | --- | --- | --- | --- | --- | --- | --- | --- |
| 0 | (CSTR1) | 1 | 2 | 3 | 4 | 5 | 6 | 7 | 8 | 9 | 10 | 11 | 12 | 13 | 14 | 15 | 16 | 17 | 18 | 19 | 20 |
| 1 | Reversal | 1 | -2 | 3 | 4 | 5 | 6 | 7 | 8 | 9 | 10 | 11 | 12 | 13 | 14 | 15 | 16 | 17 | 18 | 19 | 20 |
| 2 | Reversal | 1 | -2 | 3 | 4 | 5 | 6 | 7 | 8 | 9 | 10 | 11 | 12 | 13 | 14 | 15 | 16 | 17 | 18 | -19 | 20 |
| 3 | Reversal | 1 | -2 | 3 | 4 | 5 | 6 | 7 | 8 | -15 | -14 | -13 | -12 | -11 | -10 | -9 | 16 | 17 | 18 | -19 | 20 |
| 4 | Reversal | 1 | -2 | 3 | 4 | 5 | 6 | 7 | 15 | -8 | -14 | -13 | -12 | -11 | -10 | -9 | 16 | 17 | 18 | -19 | 20 |
| 5 | Reversal | 1 | -2 | 3 | 4 | 5 | 6 | 7 | 13 | 14 | 8 | -15 | -12 | -11 | -10 | -9 | 16 | 17 | 18 | -19 | 20 |
| 6 | Reversal | 1 | -2 | 3 | 4 | 5 | 6 | 7 | 13 | 14 | 12 | 15 | -8 | -11 | -10 | -9 | 16 | 17 | 18 | -19 | 20 |
| 7 | Reversal | 1 | -2 | 3 | 4 | 5 | 6 | 7 | 13 | 11 | 8 | -15 | -12 | -14 | -10 | -9 | 16 | 17 | 18 | -19 | 20 |
| 8 | Reversal | 1 | -2 | 3 | 4 | 5 | 6 | 10 | 14 | 12 | 15 | -8 | -11 | -13 | -7 | -9 | 16 | 17 | 18 | -19 | 20 |
| 9 | Reversal | 1 | -2 | 3 | 4 | 5 | -16 | 9 | 7 | 13 | 11 | 8 | -15 | -12 | -14 | -10 | -6 | 17 | 18 | -19 | 20 |
| 10 | Reversal | 1 | -2 | 3 | 4 | -17 | 6 | 10 | 14 | 12 | 15 | -8 | -11 | -13 | -7 | -9 | 16 | -5 | 18 | -19 | 20 |
| 11 | Reversal (MBR1b) | 1 | -2 | 3 | 5 | -16 | 9 | 7 | 13 | 11 | 8 | -15 | -12 | -14 | -10 | -6 | 17 | -4 | 18 | -19 | 20 |

Note: the minus sign in front of LCB numbers indicates that it is orientated into the direction opposite to the genome CSTR1.

Table S4: List of the 20 locally collinear blocks (LCBs) of the genome of “*Ca.* Kuenenia stuttgartiensis” strain CSTR1 after Mauve alignment with MBR1b

| LCB | CSTR1 |  | MBR1b | | | Gene content between the LCB in the current row and the LCB in the row below in strain CSTR1 | |  |
| --- | --- | --- | --- | --- | --- | --- | --- | --- |
|  | start | end | start | | end | |  |  |
| 1 | 1 | 975700 | 1 | 1005507 | | KsCSTR_10760 (transposase) | | |
| 2 | 975705 | 1099939 | 1131954 | 1007674 | | CRISPR upstream, 3 putative transposases downstream | | |
| 3 | 1099943 | 1107969 | 1131962 | 1141229 | | Gap contains 2 transposases | | |
| 4 | 1111280 | 1266103 | 3861403 | 3702476 | | KsCSTR_14130 (transposase) | | |
| 5 | 1265021 | 1848062 | 1151002 | 1689719 | | Gap contains 2 transposases | | |
| 6 | 1852741 | 1882237 | 3026904 | 2997613 | | Gap contains 3 putative transposases | | |
| 7 | 1892681 | 1923098 | 2264837 | 2291535 | | Gap is within a genomic island of 5 transposases | | |
| 8 | 1923145 | 1948499 | 2564770 | 2583012 | | Gap contains 1 putative transposase, another 2 transposases nearby | | |
| 9 | 1952829 | 2064810 | 2142299 | 2257395 | | - | | |
| 10 | 2064814 | 2188700 | 2983145 | 2867927 | | KsCSTR_24770 (DnaA) | | |
| 11 | 2187139 | 2389098 | 2346753 | 2543776 | | 9 transposases in the upstream of the gap | | |
| 12 | 2389565 | 2423369 | 2846916 | 2811400 | | - | | |
| 13 | 2424147 | 2467242 | 2303689 | 2346646 | | KsCSTR_28100 (DnaA) | | |
| 14 | 2467025 | 2485120 | 2869813 | 2854376 | | Four transposases nearby | | |
| 15 | 2485118 | 2673299 | 2802994 | 2598325 | | Inside a genomic island of 5 transposases | | |
| 16 | 2673225 | 3148954 | 2136383 | 1692718 | | Gap contains 2 transposases | | |
| 17 | 3153473 | 3809115 | 3027869 | 3699360 | | KsCSTR_43240 (transposase) | | |
| 18 | 3809763 | 3872676 | 3869724 | 3934077 | | Gap contains 1 putative transposase, another 2 transposases downstream | | |
| 19 | 3874984 | 3927021 | 3987692 | 3943047 | | Gap contains 2 transposases | | |
| 20 | 3932626 | 4334932 | 3988130 | 4406153 | |  | | |

Note: the LCBs have the same numbering as shown in Figure 1

Table S5: CRISPR elements in the anammox genomes KUST, MBR1 and CSTR1.

| Element | CRISPR Id  / Cas Type | Start | End | # Spacer / Genes | Repeat consensus / Cas genes |
| --- | --- | --- | --- | --- | --- |
| CRISPR elements in strain KUST (all in contig71) | | | | | |
| CRISPR | KUST_1 | 624472 | 629824 | 80 | GTTTTCATTCTACCTATGAGGAATTGAAAC (reverse complemented) |
| Cas cluster | CAS-TypeIB_1 | 629963 | 639422 | 7 | Cas2_0_I-II-III-V, Cas1_0_I-II-III-V, Cas4_0_I-II, Cas3_0_I, Cas5_1_IB, Cas7_2_IB, Cas6_0_I-III |
| Cas cluster | CAS-TypeIIIA_1 | 1438988 | 1447239 | 6 | Cas6_0_I-III, Cas10_0_IIIA, Csm2_0_IIIA, Csm3_1_IIID, Csm4_0_IIIA, Csm5_0_IIIA |
| CRISPR | KUST_2 | 1440247 | 1440574 | 4 | AATCATTGACCTGATGTAGAAGGGAT |
| CRISPR | KUST_3 | 1453270 | 1453903 | 8 | GTGGAAATCATTGACCTGATGTAGAAGGGATTGAGAC |
| CRISPR | KUST_4 | 1697022 | 1698279 | 19 | GTCTCATTGCACCTATAAGGAATTGAAAC |
| CRISPR | KUST_5 | 2057235 | 2057335 | 1 | GCTTGTCATTGCAGGGGTCTCGGCTTGTCATTGC |
| CRISPR elements in genome MBR1b | | | | | |
| CRISPR | MBR1b_1 | 46833 | 46908 | 1 | AGTCAATGGGGTCAAACCTAACCTA |
| CRISPR | MBR1b_2 | 111750 | 111822 | 1 | CTATAATAATTACACCCCTTCGGG |
| CRISPR | MBR1b_3 | 1848213 | 1848696 | 7 | GTCTCATTGCACCTATAAGGAATTGAAAC |
| CRISPR | MBR1b_4 | 1850805 | 1851871 | 16 | GTCTCATTGCACCTATAAGGAATTGAAAC |
| CRISPR | MBR1b_5 | 1853916 | 1854911 | 15 | GTCTCATTGCACCTATAAGGAATTGAAAC |
| CRISPR | MBR1b_6 | 1856455 | 1857003 | 8 | GTCTCATTGCACCTATAAGGAATTGAAAC |
| Cas cluster | CAS-TypeIIIA_1 | 2235811 | 2246522 | 6 | Cas6_0_I-III, Cas10_0_IIIA, Csm2_0_IIIA, Csm3_0_IIIA, Csm4_0_IIIA, Csm5_0_IIIA |
| CRISPR | MBR1b_7 | 2237065 | 2237849 | 10 | GTGGAAATCATTGACCTGATGTAGAAGGGATTGAGAC |
| CRISPR | MBR1b_8 | 2252552 | 2253195 | 8 | GTGGAAATCATTGACCTGATGTAGAAGGGATTGAGAC |
| CRISPR | MBR1b_9 | 2979462 | 2979572 | 1 | AGACCCTCGCAATTACAAGCGGAGAGACCCTCGCAATTACAA |
| Cas cluster | CAS-TypeIB_1 | 3773611 | 3783070 | 7 | Cas6_0_I-III, Cas7_2_IB, Cas5_1_IB, Cas3_0_I, Cas4_0_I-II, Cas1_0_I-II-III-V, Cas2_0_I-II-III-V |
| CRISPR | MBR1b_10 | 3783209 | 3790299 | 106 | GTTTTCATTCTACCTATGAGGAATTGAAAC |
| CRISPR elements in strain CSTR1 | | | | | |
| CRISPR | CSTR1_1 | 109398 | 109470 | 1 | CTATAATAATTACACCCCTTCGGG |
| CRISPR | CSTR1_2 | 1085339 | 1085446 | 1 | AATGATTGCAAATGTGCATGTCAC |
| CRISPR | CSTR1_3 | 1173442 | 1183846 | 156 | GTTTTCATTCTACCTATGAGGAATTGAAAC (reverse complemented) |
| Cas cluster | CAS-TypeIB_1 | 1183985 | 1193250 | 7 | Cas2_0_I-II-III-V, Cas1_0_I-II-III, Cas4_0_I-II, Cas3_0_I, Cas5_1_IB, Cas7_2_IB, Cas6_0_I-III |
| Cas cluster | CAS-TypeIIIA_1 | 2044971 | 2053123 | 6 | Cas6_0_I-III, Cas10_0_IIIA, Csm2_0_IIIA, Csm3_0_IIIA, Csm4_0_IIIA, Csm5_0_IIIA |
| CRISPR | CSTR1_4 | 2046230 | 2046482 | 3 | AATCATTGACCTGATGTAGAAGGGAT |
| CRISPR | CSTR1_5 | 2059154 | 2059926 | 10 | GTGGAAATCATTGACCTGATGTAGAAGGGATTGAGAC |
| CRISPR | CSTR1_6 | 2952952 | 2955703 | 42 | GTCTCATTGCACCTATAAGGAATTGAAAC (reverse complemented) |

Note: Repeat consensus sequences were colorized to visualize identical sequences in different genomes.

Table S6: Comparison of the large CRISPR locus near the type I-B CRISPR-Cas cluster in the three studied anammox genomes.

| Strain | KUST | MBR1b | CSTR1 |  |
| --- | --- | --- | --- | --- |
| Repeat consensus | GTTTTCATTCTACCTATGAGGAATTGAAAC | | | |
| Coordinates (bp) | 629,824-624,472 in contig71 | 3,783,209-3,790,299 (2,969,923-2,962,833 in MBR1) | 1,183,846-1,173,442 |  |
| Total length (bp) | 5,353 | 7,091 | 10,405 |  |
| # Spacers | 80 | 106 | 156 |  |
| # Spacers identical with only one of the other two strains | 16 | 26 | 26 |  |
| # Spacers identical among all three strains | 45 | | | |
| # Unique spacers (unique spacers near Cas genes) | 19 (16) | 35 (28) | 85 (80) |  |

Table S7: Protein-coding genes of “*Ca.* Kuenenia stuttgartiensis” strain CSTR1 and their abundance in the proteome. Homologous genes in the genome of strain MBR1 and KUST (including six-frame translation) are listed.

See the file TableS7.tsv

Note:

Explanation to columns:

1. Rank: the ranking of proteins based on the abundance in the whole proteome (from highest to lowest).
2. Abundance: abundance of the proteins in the whole proteome (as percent of the total amount of detected proteins in the respective samples) average of triplicates. n.d.: not detected in the proteome. d.: detected but not quantified.
3. Standard deviation (%): standard deviation of the protein abundances in the triplicate analyses.
4. KUST/MBR1 homolog: homologous genes in the genome of strain MBR1 and KUST to the corresponding gene in strain CSTR1. (six_frame): the gene was not found in the genome annotation of KUST or MBR1, but was found in the corresponding six-frame translation of the genome sequence.
5. KUST/MBR1 homolog identity (%): sequence identity of translated amino acid sequences between the homolog and the gene in strain CSTR1.

Table S8: Transposase genes and their classification in the anammox genomes KUST, MBR1 and CSTR1.

| Group | Count:  complete (remnant) | | | Total | Representative | Length (aa) | Classification |
| --- | --- | --- | --- | --- | --- | --- | --- |
|  | KUST | MBR1 | CSTR1 |  |  |  |  |
| 1 | 22 (10) | 17 (16) | 24 (12) | 101 | KsCSTR_02410 | 445 | IS1380-like element ISCku8 family transposase |
| 2 | 11 (4) | 15 (10) | 18 (7) | 65 | KsCSTR_01740 | 560 | IS1634 family transposase |
| 3 | 13 (6) | 10 (14) | 11 (8) | 62 | KsCSTR_02290 | 345 | IS630 family transposase |
| 4 | 11 (5) | 7 (3) | 10 (11) | 59 | KsCSTR_25660 | 463 | ISAs1 family transposase |
| 5 | 13 (2) | 20 (3) | 12 (0) | 50 | KsCSTR_10760 | 367 | IS4-like element ISCku3 family transposase |
| 6 | 8 (2) | 7 (2) | 8 (7) | 34 | KsCSTR_00210 | 338 | IS110 family transposase |
| 7 | 7 (3) | 10 (3) | 8 (3) | 34 | KsCSTR_14420 | 755 | IS4 family transposase |
| 8 | 9 (3) | 4 (5) | 9 (4) | 34 | KsCSTR_34150 | 640 | IS1634 family transposase |
| 9 | 4 (4) | 5 (4) | 5 (5) | 27 | KsCSTR_25380 | 295 | ISAzo13 family transposase |
| 10 | 6 (1) | 9 (0) | 8 (1) | 25 | KsCSTR_16220 | 627 | IS1634 family transposase |
| 11 | 7 (2) | 1 (2) | 6 (2) | 20 | KsCSTR_02530 | 458 | IS4-like element ISCku2 family transposase |
| 12 | 5 (2) | 7 (1) | 4 (1) | 20 | KsCSTR_23980 | 418 | ISL3 family transposase |
| 13 | 0 (0) | 20 (0) | 0 (0) | 20 | KSMBR1_2125 | 578 | IS1634 family transposase |
| 14 | 2 (3) | 4 (3) | 3 (4) | 19 | KsCSTR_11400 | 457 | IS701 family transposase |
| 15 | 4 (2) | 4 (1) | 4 (2) | 17 | KSMBR1_1977 | 342 | Rpn family recombination-promoting nuclease |
| 16 | 4 (3) | 0 (4) | 3 (3) | 17 | KsCSTR_29960 | 467 | IS4-like element ISCku4 family transposase |
| 17 | 6 (0) | 0 (4) | 7 (0) | 17 | kuste2492 | 360 | transposase |
| 18 | 4 (1) | 4 (1) | 4 (2) | 16 | KsCSTR_07160 | 492 | ISNCY-like element ISCku10 family transposase |
| 19 | 5 (1) | 2 (1) | 5 (1) | 15 | KsCSTR_16670 | 141 | IS200/IS605 family transposase |
| 20 | 1 (2) | 1 (3) | 3 (3) | 13 | KsCSTR_00240 | 507 | IS1380-like element ISCku9 family transposase |
| 21 | 3 (0) | 3 (4) | 3 (0) | 13 | KsCSTR_31040 | 473 | IS66-like element ISCku5 family transposase |
| 22 | 3 (0) | 4 (0) | 4 (1) | 12 | KsCSTR_04540 | 456 | IS66-like element ISCku7 family transposase |
| 23 | 5 (0) | 2 (0) | 5 (0) | 12 | KsCSTR_44030 | 182 | winged helix-turn-helix domain-containing protein |
| 24 | 0 (4) | 1 (3) | 0 (3) | 11 | KSMBR1_1649 | 474 | IS4 family transposase |
| 25 | 3 (0) | 3 (0) | 3 (0) | 9 | KsCSTR_25530 | 589 | transposase |
| 26 | 2 (3) | 1 (1) | 0 (2) | 9 | kuste3242 | 344 | ISAzo13 family transposase |
| 27 | 4 (0) | 0 (0) | 3 (1) | 8 | kustc0925 | 175 | IS630 family transposase |
| 28 | 0 (2) | 1 (2) | 1 (2) | 8 | KsCSTR_28280 | 153 | transposase |
| 29 | 2 (0) | 0 (2) | 0 (4) | 8 | kuste4420 | 251 | transposase istB |
| 30 | 1 (4) | 0 (1) | 0 (2) | 8 | kuste4419 | 499 | transposase istA |
| 31 | 2 (2) | 1 (0) | 2 (0) | 7 | KsCSTR_24070 | 344 | ISL3 family transposase |
| 32 | 0 (2) | 1 (2) | 0 (2) | 7 | KSMBR1_2732 | 388 | ISAzo13 family transposase |
| 33 | 2 (0) | 2 (0) | 2 (0) | 6 | kuste2652 | 129 | ISAs1 family transposase |
| 34 | 2 (0) | 2 (0) | 1 (0) | 5 | KsCSTR_30880 | 239 | transposase |
| 35 | 1 (0) | 2 (0) | 2 (0) | 5 | KsCSTR_09930 | 177 | transposase |
| 36 | 1 (0) | 1 (1) | 1 (0) | 4 | KSMBR1_0249 | 253 | transposase |
| 37 | 0 (0) | 2 (0) | 1 (0) | 3 | KSMBR1_3403 | 293 | transposase |
| 38 | 0 (0) | 1 (0) | 2 (0) | 3 | KsCSTR_08490 | 104 | transposase |

Note: Genes are considered remnants of transposase genes when they are <80% in length of the representative transposase gene.

Table S9: List of transposase genes and their classification in the anammox genomes KUST, MBR1 and CSTR1.

See the file TableS9.tsv

Note: Classification is based on representative sequences in Table S8.

Table S10: Abundances of peptides from two highly similar hydrazine dehydrogenases KsCSTR_46980 and KsCSTR_11820 in the proteome of “*Ca.* Kuenenia stuttgartiensis” strain CSTR1.

| Peptide sequence | # PSMs | Position in KsCSTR_46980 | Position in KsCSTR_11820 | Abundances in triplicate samples | |  | |
| --- | --- | --- | --- | --- | --- | --- | --- |
| HQFDPAVAR | 9 | [294-302] | [234-242] | 1.76E+08 | 2.07E+08 | | 1.79E+08 |
| DEVRPSNPIK | 12 | [164-173] | [104-113] | 1.58E+08 | 1.65E+08 | | 1.51E+08 |
| TGEWLDQLTGPYIVK | 38 | [565-579] | [505-519] | 1.58E+08 | 1.50E+08 | | 1.61E+08 |
| WDTEQFDFSK | 17 | [336-345] | [276-285] | 1.43E+08 | 1.28E+08 | | 1.15E+08 |
| IGAYHDGEAYGGTTGESGEFR | 26 | [466-486] | - | 1.28E+08 | 1.63E+08 | | 1.48E+08 |
| DLCPDWSGQHIWSLK | 26 | [451-465] | [391-405] | 1.17E+08 | 1.12E+08 | | 1.02E+08 |
| VGISWQPEQFWK | 15 | [553-564] | [493-504] | 8.66E+07 | 1.04E+08 | | 9.33E+07 |
| VFFSGLK | 25 | [117-123] | [57-63] | 8.41E+07 | 5.75E+07 | | 6.60E+07 |
| WLVNVK | 15 | [532-537] | [472-477] | 6.92E+07 | 4.82E+07 | | 5.03E+07 |
| DHRDWEAYDIGLHGTVYQVNK | 13 | [315-335] | [255-275] | 3.91E+07 | 5.46E+07 | | 3.45E+07 |
| DWEAYDIGLHGTVYQVNK | 20 | [318-335] | [258-275] | 3.60E+07 | 4.75E+07 | | 4.53E+07 |
| TIFDLCPDPGWLDTHHAPAEEVEYIER | 19 | [585-611] | [525-551] | 3.35E+07 | 5.66E+07 | | 4.82E+07 |
| ENLQAMDESVKDASLK | 7 | [413-428] | [353-368] | 3.32E+07 | 2.51E+07 | | 1.91E+07 |
| WDTEQFDFSKK | 5 | [336-346] | [276-286] | 2.63E+07 | 2.09E+07 | | 1.98E+07 |
| MSNVTDVER | 4 | [487-495] | [427-435] | 2.23E+07 | 3.02E+07 | | 2.39E+07 |
| ELGITAGSHSAHHHESGHDPAAR | 9 | [615-637] | - | 1.78E+07 | 1.85E+07 | | 1.98E+07 |
| YYIPDYMYNRDEVRPSNPIK | 6 | [154-173] | [94-113] | 1.70E+07 | 1.50E+07 | | 9.53E+06 |
| VAEDLLIDGVLDPMPK | 11 | [435-450] | [375-390] | 1.26E+07 | 1.45E+07 | | 1.26E+07 |
| YYIPDYMYNR | 3 | [154-163] | [94-103] | 1.12E+07 | 8.76E+06 | | 6.80E+06 |
| ENLQAMDESVK | 7 | [413-423] | [353-363] | 9.46E+06 | 9.60E+06 | | 8.38E+06 |
| KVGISWQPEQFWK | 6 | [552-564] | [492-504] | 7.02E+06 | 1.35E+07 | | 1.20E+07 |
| LTMPSSK | 3 | [222-228] | [162-168] | 5.60E+06 | 4.72E+06 | | 3.84E+06 |
| MSNVTDVER | 11 | [487-495] | [427-435] | 3.53E+06 | 1.75E+06 | | 2.10E+06 |
| LCFESVGYFQTYIYK | 67 | [496-510] | [436-450] | 2.70E+06 | 5.89E+06 | | 6.50E+06 |
| ASIVYTSMGMSMADR | 9 | [372-386] | [312-326] | 2.19E+06 | 2.11E+06 | | 1.25E+06 |
| DHRDWEAYDIGLHGTVYQVNKWDTEQFDFSK | 2 | [315-345] | [255-285] | 1.84E+06 | 6.07E+06 | | 2.94E+06 |
| VAEDLLIDGVLDPMPK | 17 | [435-450] | [375-390] | 1.50E+06 | 1.33E+06 | | 7.46E+05 |
| ASIVYTSMGMSMADR | 2 | [372-386] | [312-326] | 1.25E+06 | 1.05E+06 | | 1.34E+06 |
| DWEAYDIGLHGTVYQVNKWDTEQFDFSK | 6 | [318-345] | [258-285] | 5.29E+05 | 1.91E+06 | | 1.57E+06 |
| HQFDPAVARR | 1 | [294-303] | [234-243] | 5.13E+05 | 5.65E+05 | | 5.01E+05 |
| ENLQAMDESVK | 3 | [413-423] | [353-363] | 4.71E+05 | 4.48E+05 | | 3.33E+05 |
| ENLQAMDESVKDASLKYR | 1 | [413-430] | [353-370] | 2.46E+05 | 2.14E+05 | | 1.76E+05 |
| ETFKVAEDLLIDGVLDPMPK | 4 | [431-450] | [371-390] | 1.11E+05 | 1.99E+05 | | 2.22E+05 |
| YMGYPKDAQR | 1 | [126-135] | [66-75] | - | 2.03E+05 | | - |

Table S11: Detection of three nitrite reductase gene candidates in the proteome of “*Ca.* Kuenenia stuttgartiensis” strain CSTR1 over time.

| Sampling time | # Sample | Reactor   / wall material | HRT (d) | Digestion | # Total PSM ^a^ | # PSM1 ^b^ | # PSM2 ^c^ | # PSM3 ^d^ | Comment |
| --- | --- | --- | --- | --- | --- | --- | --- | --- | --- |
| 2017.01 | 4 | 2L / glass | ∞ | Solution | 9114 | 80 | 87 | 37 | Reactor temperature rose to 38.6 °C 7 days before sampling, after which high nitrite (>3 mM) and low anammox activity were observed. No reactor flow. |
| 2019.03 | 4 ^e^ | 1L / glass | 3 | Gel | 52936 | 0 | 299 | 244 |  |
| 2019.04 | 4 | 30L / polyethylene | 8 | Solution | 3734 | 0 | 58 | 26 |  |
| 2019.07 | 4 ^e^ | 30L / polyethylene | 15 | Gel | 157166 | 81 | 574 | 601 |  |
| 2019.10 | 3 | 1L / glass | 3 | Solution | 20800 | 0 | 133 | 57 |  |
| 2020.01 | 2 | 30L / polyethylene | >30 | Solution | 5659 | 12 | 53 | 26 | High HRT for > 30 days |

^a^ Total PSM: number of total PSMs of all identified proteins in the respective dataset.

^b^ # PSM1: number of PSMs of KsCSTR_33370 (homolog of kuste4136 and KSMBR1_0452), annotated as nitrite reductase (*nirS*)

^c^ # PSM2: number of PSMs of KsCSTR_49490 (homolog of kustc0458 and KSMBR1_2163), annotated as putative hydroxylamine oxidoreductase

^d^ # PSM3: number of PSMs of KsCSTR_29630 (homolog of kuste4574 and KSMBR1_3792), annotated as putative hydroxylamine oxidoreductase

^e^ For protein samples that were separated by blue native PAGE, the sample numbers refer to the number of gel lanes. Each gel lane was excised into 13 gel slices, and in-gel digestion was performed before shot-gun proteomics.

Table S12: List of peptides detected in the S-layer protein KsCSTR_09970 in a series of “*Ca.* Kuenenia stuttgartiensis” strain CSTR1 samples.

| Position | Sequence | Length | # Missed cleavages | # PSMs |
| --- | --- | --- | --- | --- |
| 256-284 | VQVIDQDSATGDALTGATASNIILVETGK | 29 | 0 | 1650 |
| 932-958 | VVFASNSSSVTSTDTSNATTSNTTHFK | 27 | 0 | 1168 |
| 234-253 | EVIGLQDGFTTGLASAGSSR | 20 | 0 | 941 |
| 1108-1125 | SVADGVVTVTYQENSPAR | 18 | 0 | 834 |
| 563-582 | VAGYSNSSFVIDLQHQDGSR | 20 | 0 | 751 |
| 1217-1230 | IQSQTGDTITATFK | 14 | 0 | 533 |
| 1480-1501 | GDSEDVIVLVTGDNECPAQGVK | 22 | 0 | 407 |
| 394-418 | DTNDEVSLFGNPTSNTSAYLPSSTR | 25 | 0 | 302 |
| 219-233 | ITVVDPNVNLNPNLK | 15 | 0 | 290 |
| 786-799 | TWVQVVGNDMEPNR | 14 | 0 | 267 |
| 992-1027 | VVSGDTVTIYYNDSPSASNENNLQNLTTVSIVTSAR | 36 | 0 | 262 |
| 1526-1534 | TGQATFTVK | 9 | 0 | 252 |
| 139-161 | LITGGLGSSAPGNAANGTDGVLK | 23 | 0 | 241 |
| 1126-1134 | DVTAQVSTK | 9 | 0 | 237 |
| 517-541 | TSVDINDFFAITVVDGNLNTSSTAR | 25 | 0 | 213 |
| 1093-1104 | TGASTDSTTTPK | 12 | 0 | 189 |
| 775-785 | MQNTGFYTPWK | 11 | 0 | 174 |
| 1557-1563 | VNVSLSK | 7 | 0 | 159 |
| 590-613 | ISSTDGSLIWVVPNSLSDSTYGFR | 24 | 0 | 158 |
| 751-757 | ISDGIYK | 7 | 0 | 155 |
| 43-56 | DHYLPQLGTSDYDR | 14 | 0 | 150 |
| 422-432 | LIDGANYCLVK | 11 | 0 | 140 |
| 1028-1035 | EGTLSLSK | 8 | 0 | 113 |
| 1514-1522 | IKVTPASQK | 9 | 1 | 83 |
| 1505-1511 | KLTSANK | 7 | 1 | 62 |
| 1516-1522 | VTPASQK | 7 | 0 | 54 |
| 761-774 | EVTGVSGTTLSVTK | 14 | 0 | 53 |
| 1456-1479 | ICGEPGEVEASNATVTPDILDLVK | 24 | 0 | 36 |
| 800-810 | ADTISGTQLFR | 11 | 0 | 30 |
| 1135-1176 | NFGAVLDITDDTVALGGSAVVSLYDPESNTSIDTANIVGTVR | 42 | 0 | 27 |
| 751-760 | ISDGIYKEFR | 10 | 1 | 26 |
| 561-582 | LKVAGYSNSSFVIDLQHQDGSR | 22 | 1 | 16 |
| 388-418 | VVVGIRDTNDEVSLFGNPTSNTSAYLPSSTR | 31 | 1 | 13 |
| 219-253 | ITVVDPNVNLNPNLKEVIGLQDGFTTGLASAGSSR | 35 | 1 | 12 |
| 1523-1534 | TDRTGQATFTVK | 12 | 1 | 11 |
| 1108-1134 | SVADGVVTVTYQENSPARDVTAQVSTK | 27 | 1 | 10 |
| 959-970 | LVETAVNSGTFK | 12 | 0 | 7 |
| 1093-1107 | TGASTDSTTTPKTIR | 15 | 1 | 4 |
| 1526-1536 | TGQATFTVKAK | 11 | 1 | 4 |
| 1514-1525 | IKVTPASQKTDR | 12 | 2 | 3 |
| 1546-1556 | FGVKGVKVTPK | 11 | 2 | 2 |
| 1553-1563 | VTPKVNVSLSK | 11 | 1 | 2 |
| 1105-1125 | TIRSVADGVVTVTYQENSPAR | 21 | 1 | 1 |
| 419-432 | IFKLIDGANYCLVK | 14 | 1 | 1 |
| 254-284 | VRVQVIDQDSATGDALTGATASNIILVETGK | 31 | 1 | 1 |
| 590-624 | ISSTDGSLIWVVPNSLSDSTYGFRDPLTPGSSSFK | 35 | 1 | 1 |

References:

1. van Teeseling MCF, Maresch D, Rath CB, Figl R, Altmann F, Jetten MSM, Messner P, Schäffer C, van Niftrik L: The s-layer protein of the anammox bacterium *Kuenenia stuttgartiensis* is heavily o-glycosylated. Frontiers in Microbiology 2016, 7(1721).

2. Hu Z, Speth D, Francoijs K-J, Quan Z-X, Jetten M: Metagenome analysis of a complex community reveals the metabolic blueprint of anammox bacterium “*Candidatus* Jettenia asiatica”. Frontiers in Microbiology 2012, 3:366.

3. van de Vossenberg J, Woebken D, Maalcke WJ, Wessels HJCT, Dutilh BE, Kartal B, Janssen-Megens EM, Roeselers G, Yan J, Speth D *et al*: The metagenome of the marine anammox bacterium ‘*Candidatus* Scalindua profunda’ illustrates the versatility of this globally important nitrogen cycle bacterium. Environ Microbiol 2013, 15(5):1275-1289.

4. Tesler G: GRIMM: genome rearrangements web server. Bioinformatics 2002, 18(3):492-493.
